# An Expanded View of RNA Modification with Carbohydrate-Based Metabolic Probes

**DOI:** 10.1021/jacsau.5c00249

**Published:** 2025-05-05

**Authors:** Madoka E. Hazemi, Michael B. Geeson, Felix M. Müller, Sigitas Mikutis, Anton J. Enright, Gonçalo J. L. Bernardes

**Affiliations:** † Yusuf Hamied Department of Chemistry, 2152 University of Cambridge, Lensfield Road, Cambridge CB2 1EW, United Kingdom; ‡ Department of Pathology, 2152 University of Cambridge, Tennis Court Road, Cambridge CB2 1QP, United Kingdom; § GiMM - Gulbenkian Institute for Molecular Medicine, Avenida Prof. Egas Moniz, 1649-028 Lisboa, Portugal; ∥ Translational Chemical Biology Group, Spanish National Cancer Research Centre (CNIO), Madrid 28029, Spain

**Keywords:** RNA glycosylation, metabolic labeling, glycosidase treatment, RNA sequencing

## Abstract

The discovery of glycosylated RNA (glycoRNA) revealed an unexpected association between glycans and RNA. This discovery, facilitated by the metabolic carbohydrate reporter per-O-acetylated N-azidoacetyl-mannosamine, underscores the potential for biological roles of previously unknown RNA modifications. Our study builds upon the detection of glycoRNA with other metabolic probes such as per-O-acetylated N-azidoacetyl-glucosamine, N-azidoacetyl-galactosamine, and 6-azidofucose. This broader analysis uncovered potential new glycosylated transcripts that warrant further study. However, our results also revealed unexpected resilience of the unidentified carbohydrate–RNA linkage to enzymatic cleavages by glycosidases. Therefore, we investigated nonenzymatic formation of carbohydrate–RNA linkages in vitro using per-acetylated probes. From these studies, we draw two main conclusions. First, signals arising from metabolic incorporation using acetylated carbohydrate probes represent the main source of detectable glycoRNA species. Second, sequencing reveals that the most likely candidates for carbohydrate-modified RNAs are likely tRNAs. We demonstrated that an expanded repertoire of metabolic reporters can be incorporated into glycoRNA, opening new perspectives on the nature of post-transcriptionally modified RNA.

## Graphical abstract

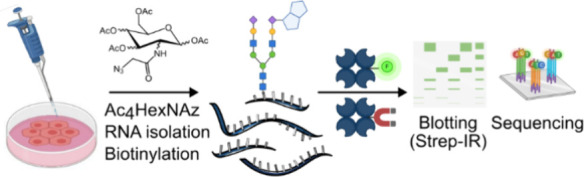

## Introduction

Glycosylation is one of the most abundant and complex post-translational modifications in biology and controls many essential cellular functions such as protein folding, stability, tracking, and immune system recognition.^1–4^ The field of glycobiology, which investigates these processes, has been significantly advanced through the use of chemical tools such as metabolic chemical reporters (MCRs).^5–7^ This technique employs sugar precursors equipped with a relatively small reactive handle, allowing them to enter the salvage pathway and mimic natural sugars, thus facilitating the probing of glycans. One notable reporter, per-O-acetylated N-azidoacetyl-mannosamine (Ac_4_ManNAz), has been extensively utilized to investigate glycans within the realm of glycoproteins due to its conversion to sialic acid azide, a common feature in most glycan termini.^8,9^ In an unexpected turn, recent work by Flynn and Bertozzi et al. used this probe to reveal the presence of glycosylated RNA (glycoRNA) on cell surfaces.^10^ Despite the prevalence of glycosylated proteins, lipids and metabolites, a direct link between glycans and RNA was previously unknown, apart from a few monosaccharide modifications on tRNAs.^11–15^ In a following study, the post-transcriptional modification 3-(3-amino-3-carboxypropyl)­uridine (acp^3^U), commonly found in tRNAs, was identified as one of the attachment sites between glycans and RNA.^16^ This approach took advantage of the chemical properties of carbohydrates, in which a terminal diol of a glycan was oxidized by periodate for subsequent enrichment and analysis of the glyco-conjugate.^17^ Apart from metabolic labeling or chemical alteration of native carbohydrates, recognition of glycans by lectins, antibodies or even aptamers are commonly used techniques in glycobiology.^18^ The discovery of glycoRNA was therefore further supported by research from Ma et al., who used sialic acid recognizing aptamers to visualize glycoRNA at the single cell level.^19^ While the exact biochemical nature and biological role of glycoRNA have yet to be fully deciphered, expanded techniques for the detection of glycoRNA are desired.

In the course of developing new methods for identifying post-transcriptional modifications,^20^ and intrigued by glycosylation of RNA, we reasoned that other established metabolic chemical reporters,^21,22^ namely per-O-acetylated N-azidoacetyl-glucosamine (Ac_4_GlcNAz), N-azidoacetyl-galactosamine (Ac_4_GalNAz) and 6-azidofucose (Ac_4_FucAz) could be incorporated into glycoRNA as well. The use of these metabolic labeling reporters enables comprehensive coverage of both extracellular and intracellular N-linked and O-linked glycans. N-acetylglucosamine and N-acetylgalactosamine core reporters are of particular interest, as they are found at the base of most mammalian O-linked glycan structures and might therefore offer an opportunity for detecting new glycoRNA transcripts.^23^ While the Ac_4_GlcNAz reporter is recognized for its potential in probing O-glycans, it has low metabolic labeling activity, likely a result of a metabolic bottleneck at the UDP-GlcNAc pyrophosphorylase step of the GlcNAc salvage pathway.^24,25^ Additionally, the labeling efficiency of Ac_4_GlcNAz can be affected by its propensity to undergo multiple pathways, including conversion to ManNAz within cells and subsequently to sialic acid azide, rendering it analogous to the Ac_4_ManNAz probe.^26^ Consequently, we employed Ac_4_GalNAz due to its ability to efficiently convert UDP-GalNAz and UDP-GlcNAz in an approximate 1:3 ratio, enabling an increased coverage for O-linked glycans.^25,27^ On the other hand, Ac_4_FucAz probe is of interest because it structurally distinguishes itself from the other hexose sugars due to its L-configuration and lack of an N-acetyl group at the 2-position as well as a hydroxyl group at the 6-position. Ac_4_FucAz exploits the fucose salvage pathway to access GDP-FucAz, followed by transfer to the termini of N-linked and O-linked glycans as L-fucose azide.^28^

The expanded view on glycoRNA by repurposing metabolic carbohydrate reporters conveniently used for glycoprotein analysis, could therefore reveal glycosylated transcripts that have not been detected by metabolic labeling with Ac_4_ManNAz or immobilization of glycoRNA on solid phases.^10,29^

## Results

### Metabolic Labeling with Per-O-Acetylated Azido Sugars

Our studies began with validating prior work on detection of glycoRNA using the azide containing metabolic probe for N-acetyl mannosamine (Ac_4_ManNAz)^10^ and further expanding the metabolic reporter scope by applying other per-O-acetylated azido sugars, e.g., Ac_4_GlcNAz, Ac_4_GalNAz and Ac_4_FucAz. HeLa cells were probed with 100 μM of the respective metabolic reporter for 24 h and RNA was extracted using the acid guanidinium thiocyanate–phenol–chloroform (AGPC) method, a technique that ensures high-yield RNA and removal of proteins and other contaminants (Figure 1A).^30,31^ Following these initial steps, RNA was subjected to strain–promoted azide–alkyne cycloaddition (SPAAC) with DBCO-biotin. RNA was optionally separated into large (>200 nts) and small (<200 nts) fractions using silica-based spin columns when needed, analyzed by agarose gel electrophoresis, transferred to a nitrocellulose membrane, and blotted with the dye Streptavidin-IR 800 (Strep-IR) stain to detect biotinylated RNA signal. We observed clear Strep-IR signal of the total RNA from HeLa cells probed with Ac_4_ManNAz, Ac_4_GlcNAz, and Ac_4_GalNAz (Figure 1B). The probe Ac_4_FucAz, on the other hand, did not produce any Strep-IR signal indicating the absence of azide sugar on the RNA. Interestingly, the Ac_4_GalNAz reporter exhibited the highest labeling intensity in Strep-IR, while Ac_4_GlcNAz displayed the lowest. This observation aligns with previous research demonstrating superior O-GlcNAcylated protein labeling by the Ac_4_GalNAz probe due to its efficient conversion to UDP-GlcNAz, contrasting the poor labeling efficiency of Ac_4_GlcNAz due to the limiting UDP-GlcNAc pyrophosphorylase step in the GlcNAc salvage pathway and its inefficient conversion to ManNAz through its multiple conversion pathways.^25,26^ However, in light of a recent study, we suggest caution when inferring quantitative conclusions from carbohydrate-based metabolic probes, as azide presence could potentially interfere with the salvage pathway, and as a result, may not provide an accurate representation of the natural sugar distribution.^32^ Therefore, the scope of our analysis is limited to detection sensitivity, rather than precise quantification, among these four different metabolic probes.

**Figure 1:**
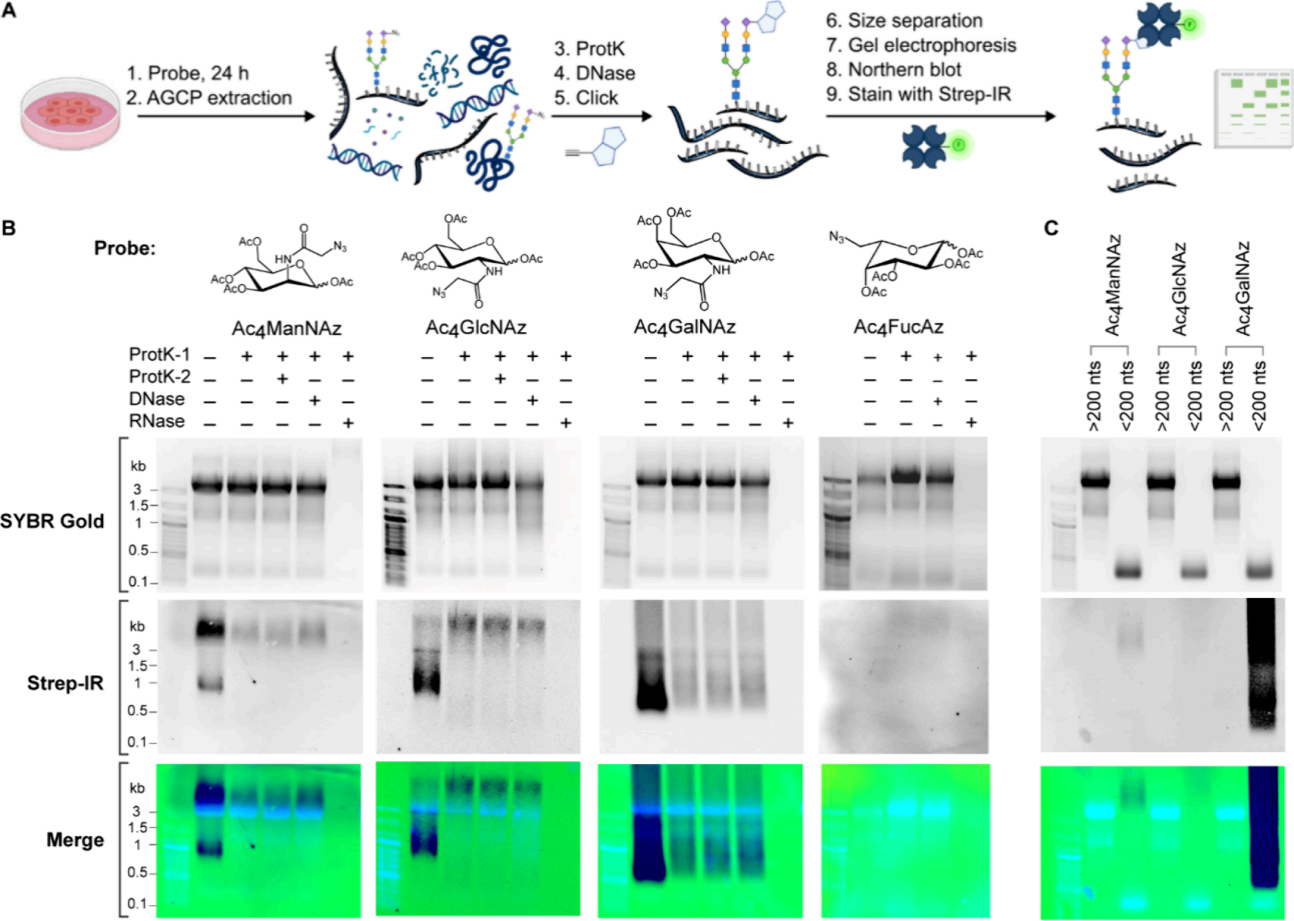
Characterization of Ac_4_ManNAz, Ac_4_GlcNAz, and Ac_4_GalNAz as metabolic probes for cellular RNA detection and analysis. (A) Schematic illustrating the process of cellular probing with sugar azide, followed by RNA extraction and purification. Post purification, the sugar azide is conjugated to DBCO-biotin or alkyne-biotin. Total RNA and glycoRNA are visualized using SYBR-Gold and streptavidin-IR800 (Strep-IR), respectively. (B) Gel electrophoresis and Strep-IR blotting results of total RNA from HeLa cells treated with 100 μM of Ac_4_ManNAz, Ac_4_GlcNAz, Ac_4_GalNAz, or Ac_4_FucAz. RNA is subjected to in vitro treatment with Prot K, Turbo DNase, or RNase cocktail (A/T1), as specified in each lane. (C) Gel electrophoresis (SYBR Gold) and Strep-IR blotting results of total RNA from HeLa cells treated with 100 μM Ac_4_ManNAz, Ac_4_GlcNAz, and Ac_4_GalNAz, followed by size separation using silica-based columns.

To ensure the signals originated from RNA and were not a consequence of protein contamination, we treated the probed biotinylated-RNA with two successive rounds of ProtK and we found no substantial change in signal intensity between the first and second treatment (Figure 1B). In addition, we noted that intense ProtK treatment at high concentrations and temperatures, even alongside a significant concentration of urea, had minimal impact on the RNA signal but was effective at removing residual proteins (Figure S1A–C). When we subjected the labeled RNA to DNase treatment, the signal persisted, however, treatment of an RNase cocktail (A and T1) and subsequent purification by silica-based spin column, using 50% ethanol for precipitation, led to the complete removal of both the total RNA and the biotinylated RNA signal in each sample (Figure 1B). These observations suggest that the signals we detected were derived from metabolically probed RNA and are henceforth referred to as ManNAz-RNA, GlcNAz-RNA and GalNAz-RNA, respectively. Following a length-based separation of RNA using a silica-based column, the biotinylated RNA signals were detected in the small RNA fractions (<200 nts) as marked by the RNA SYBR Gold signal in the low molecular weight (MW) region (Figure 1C). Intriguingly, the SYBR Gold and Strep-IR signal for all samples did not overlap; the Strep-IR signal that represents the biotinylated glycoRNA fraction migrated at a higher MW region than the SYBR Gold signal of small RNA.

To eliminate the possibility that noncovalent interactions of DBCO-biotin with RNA or its impact on the transfer to the nitrocellulose membrane were causing the observed nonoverlapping signals between SYBR Gold and Strep-IR, we employed CuAAC with alkyne-biotin treated RNA for Northern blotting, in addition to a direct visualization method on the agarose gel using alkyne-fluor 488 (Figure 2A). We observed that the shift in the Strep-IR signal from biotinylated RNA remains the same, whether using an alkyne or DBCO probe, suggesting that the DBCO moiety itself did not contribute to the observed slow migration (Figure 2B). To our surprise, the fluor 488-alkynylated small RNA signal diverged from the Strep-IR trend. It migrated alongside small RNA with an apparent molecular weight of 500 nts (Figures 2C and S1D), possibly due to the fluorophore bearing an additional negative charge. This prompted the hypothesis that the biotin functional group was responsible for the slow migration of RNA functionalized with DBCO- or alkyne-biotin. To test this hypothesis, we designed a synthetic RNA with an azide moiety in the middle of the sequence (YZ RNA) and performed a click reaction with either DBCO-biotin followed by Strep-IR blotting analysis or CuAAC with fluor 488-alkyne followed by agarose gel electrophoresis. CuSO_4_ was omitted in control samples to show that the fluor 488 signals come specifically from clicking with modified RNA and not nonspecific intercalation of the fluorophore with RNA. Interestingly, neither the biotin nor the fluor 488-RNA conjugates of YZ RNA resulted in a substantial migration to the higher MW region (Figure S1E). This suggests that the apparent migration of the signal to the higher MW region may be attributable to an effect of the glycan in combination with its conjugation probe.

**Figure 2:**
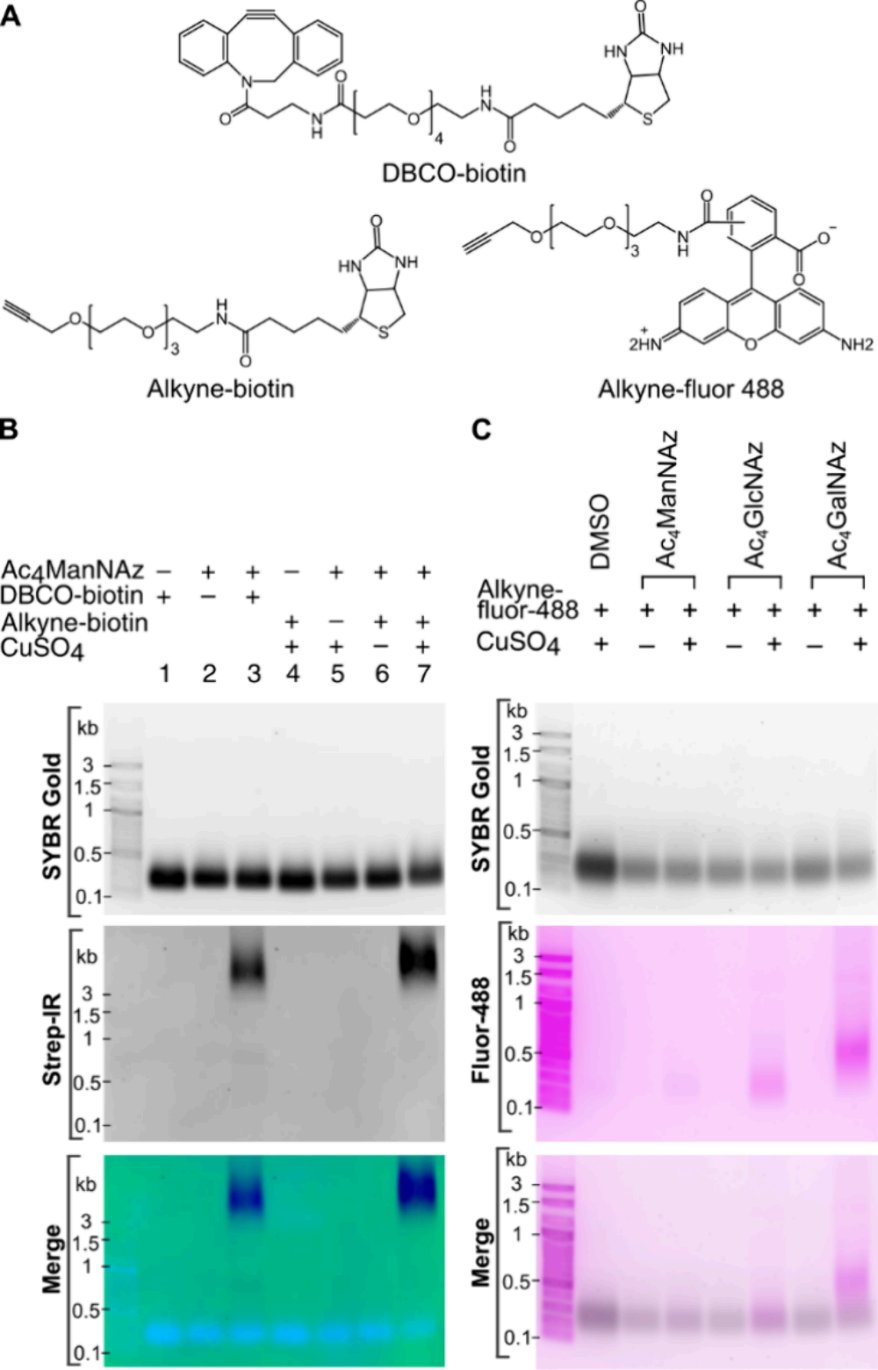
Testing different probes for glycoRNA imaging. (A) Molecular structure of DBCO-biotin, alkyne-biotin and alkyne-fluor 488 (B) Gel electrophoresis and Strep-IR blotting results of HeLa small RNA (<200 nts) probed with 100 μM Ac_4_ManNAz and subjected to either SPAAC with DBCO-biotin or CuAAC with alkyne-biotin and their respective negative controls as indicated on each lane. (C) Gel electrophoresis results of HeLa small RNA (<200 nts) probed with 100 μM Ac_4_ManNAz, Ac_4_GlcNAz, or Ac_4_GalNAz, then subjected to CuAAC with alkyne-fluor 488.

### GlycoRNA Labeling of Different Cell Lines

Expanding on this metabolic labeling approach, we investigated the presence of glycoRNA in a variety of other cancer cell types. The Ac_4_ManNAz probe was employed to maintain consistency and allow comparative analysis with the initial report.^10^ We categorized the cell lines into two distinct groups for our analysis. Group 1 comprised adherent cell lines, including an embryonic kidney cell line (HEK293T), a pancreatic cancer cell line (PANC1), a renal cell carcinoma cell line (SKRC52), and a breast cancer cell line (SKBR3) while group 2 comprised suspension cell lines, which included an acute myeloid leukemia cell line (MOLM-13), an erythroblast cell line (Hel), an acute T-cell leukemia cell line (Jurkat), and a chronic lymphocytic leukemia cell line (WA-C3CD5+). Among all the cell lines studied, SKRC52 presented the most pronounced ManNAz-RNA signal, displaying approximately ten times more intense signal relative to HeLa cells (Figures 3A and S1F). Adherent cells consistently exhibited stronger ManNAz-RNA signals than suspension cell lines (Figures 3B and S1G). There are several possibilities that could explain this variation in ManNAz-RNA signals among cell lines and across cell types.^33,7^ Some studies have suggested that initial phosphorylation of ManNAc by N-acetylglucosamine kinase and/or N-acetylmannosamine kinase is the bottleneck in the salvage pathway of sialic acid.^34,35^ Collectively, both differences in metabolism and cellular uptake can influence the efficiency of incorporation of sugar probes into glycoRNA. These findings show that glycoRNA modification is context-dependent and influenced by the cellular environment.

**Figure 3:**
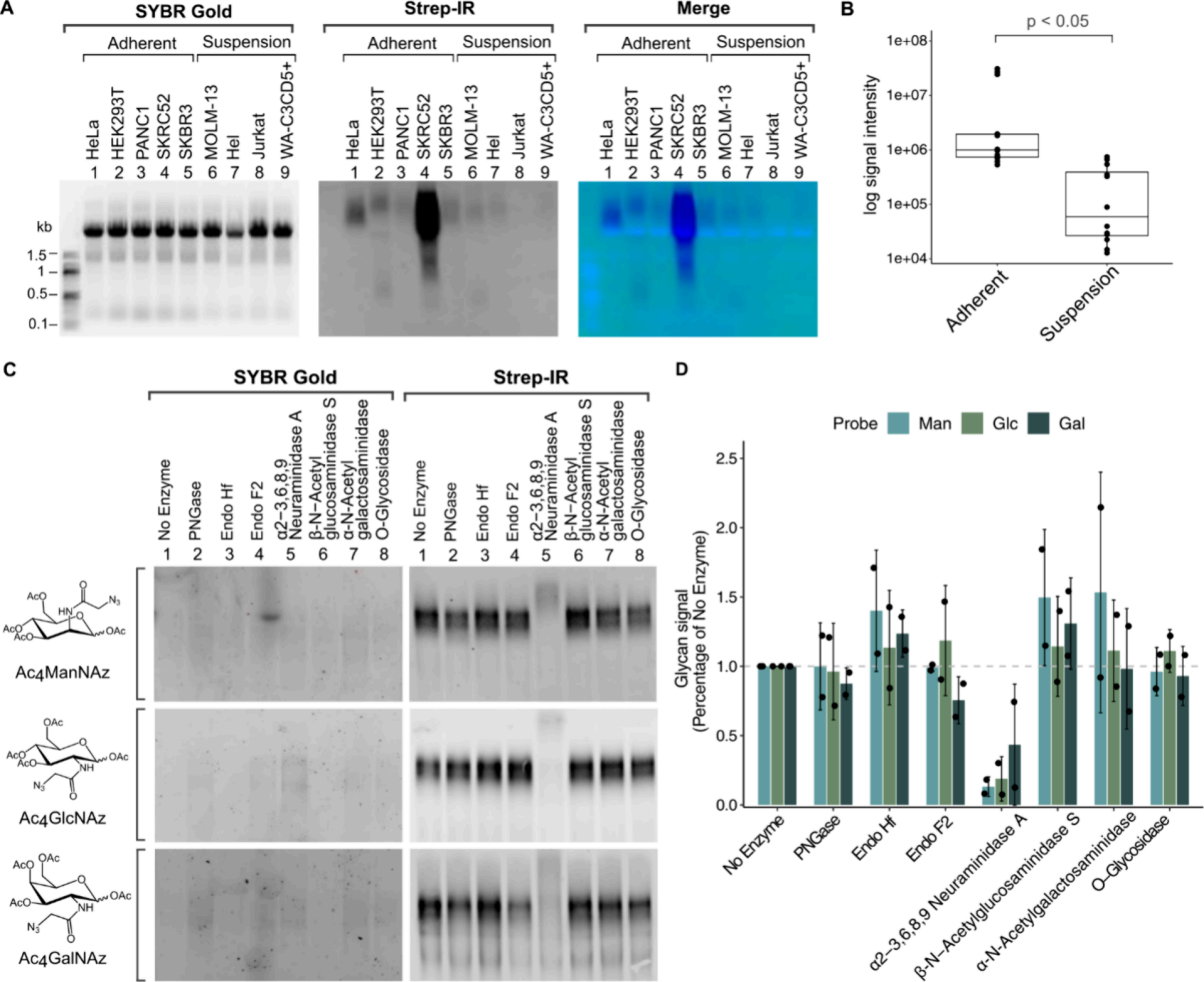
GlycoRNA distribution in various cancer cell types, the efficacy of glycoRNA release and enzymatic treatment methods and glycosidase effect on glycoRNA. (A) Gel electrophoresis (SYBR Gold) and Strep-IR blotting results of total isolated ManNAz-RNA from various cancer cells including HeLa, HEK293T, PANC1, SKRC52, SKBR3, MOLM-13, Hel, Jurkat, WA-C3CD5+ grouped by their adherent or suspension cell type. (B) Box plots of the distribution of ManNAz-RNA Strep-IR signal intensities for each cell type on a log scale. Each data point represents a biological replicate, and the central line in each box indicates the median value. Statistically significant differences (*p* < 0.05) between groups are indicated. (C) Gel electrophoresis (SYBR Gold) and Strep-IR blotting results of enriched ManNAz-, GlcNAz-, and GalNAz-RNA from HeLa cells after in vitro treatment with the indicated glycosidases for 1 h at 37 °C. (D) Bar plot of quantification of ManNAz, GlcNAz, and GalNAz-RNA Strep-IR signals following in vitro treatment with the indicated glycosidases. Each experiment was conducted in biological duplicates with each point in the graph representing an individual biological replicate. Error bars represent the standard deviation (SD) to illustrate the variability within the biological replicates.

### GlycoRNA Enrichment and Analysis Using Streptavidin Pulldown and Release Method

In addition to the visualization of glycoRNA by Strep-IR staining, the biotin-probed RNA can be purified and enriched for downstream analysis and sequencing purposes using a pulldown and release method. In this instance we expect glycoRNA to largely fail to reverse-transcribe during library-preparation, hence we are looking for significant attrition of sequencing reads between control and test sample RNA species as evidence for glycosylation. We first established a protocol that includes enrichment of biotinylated ManNAz-RNA using streptavidin beads, a stringent washing protocol to remove nonspecifically bound RNA transcripts, and release from the beads by heating at 95 °C in formamide for 2 min (Figure S2A). Formamide can be conveniently removed by dilution in water and lyophilization, and while the presence of EDTA (10 mM) appeared to enhance release of biotinylated RNA (not shown), it was omitted in our final protocol so that RNA could be obtained in pure nuclease-free water. Of note, our attempts to obtain biotinylated RNA from formamide using either silica spin columns or precipitation with glycogen were unsuccessful, a result we ostensibly attribute to poor precipitation and low quantities of glycoRNA (not shown).

To verify the efficacy of our protocol, we analyzed the enriched biotinylated ManNAz-small RNA of the two cell lines, SKRC52 and HeLa. We observed clear signals on the Strep-IR membrane for both cell lines, correlating with their respective labeling intensities before pulldown, which suggests the successful isolation and recovery of biotinylated ManNAz-small RNA without detection bias (Figure S2B). This signal was observed only for ManNAz-small RNA that had been subjected to DBCO-biotin and disappeared after RNase (A and T1) treatment, affirming the authenticity of the biotinylated ManNAz-small RNA signal. However, we observed faint signals in the low molecular weight range on an agarose gel stained by SYBR Gold that do not overlap with the Strep-IR signal at high molecular weight. This indicates either nonspecific pulldown or partial degradation of glycoRNA under the pulldown conditions. Control experiments with nonbiotinylated RNA (Figure S2B lanes 1 and 4 and S2C lane 1) exhibit neither SYBR Gold nor Strep-IR signals, confirming the absence of nonspecific interactions of RNA with streptavidin beads. To rule out potential nonspecific interactions between nonbiotinylated and biotinylated RNA that are enriched, we integrated a washing step with a 6 M urea buffer into our protocol to disrupt nonspecific base pairings. Comparative analysis posturea treatment showed no significant changes compared to the standard conditions (Figure S2C), with Strep-IR signal residing in high molecular weight and SYBR gold in low molecular weight area, ruling out any unspecific RNA pulldown and reinforcing our confidence in the affinity purification process.

### ManNAz-, GlcNAz-, and GalNAz-RNA Linkage Investigation by Glycosidase Treatment

The successful labeling of RNA with Ac_4_ManNAz, Ac_4_GlcNAz, Ac_4_GalNAz offers comprehensive means for probing the glycan landscape on RNA. The two major categories of glycans found on proteins are N- and O-glycans. Both glycan types can undergo sialylation, a modification that allows them to be tagged by the Ac_4_ManNAz probe. The Ac_4_GlcNAz probe exhibits versatility in this context as it can be converted either into sialic acid or into UDP-GlcNAz, enabling it to label both N- and O-glycans.^26^ The probe Ac_4_GalNAz also includes conversion into UDP-GalNAz or UDP-GlcNAz, both of which are precursors for O-glycan synthesis.^25^ As such, the use of these three probes, each with its specific targets and metabolic pathways, allows for comprehensive probing of the glycan landscape on RNA.

To discern whether these structures were akin to those found on glycoproteins, we treated HeLa-derived, purified ManNAz-RNA, GlcNAz-RNA, and GalNAz-RNA with a panel of endoglycosidases including PNGase, Endo Hf, Endo F2, as well as exoglycosidase enzymes such as α-2-3,6,8,9 neuraminidase A (Sialidase), β-N-acetylglucosaminidase S (GlcNase), α-N-acetylgalactosaminidase (GalNase), and O-glycosidase. Post-treatment, RNAs were purified using the pulldown and release method and analyzed via gel electrophoresis and Strep-IR blotting.

To our surprise, treatment of ManNAz-RNA, GlcNAz-RNA, and GalNAz-RNA with PNGase F, an enzyme that hydrolyses the glycosamine linkage on the asparagine side chain of a wide variety of glycoproteins and N-glycans,^36^ had no impact on the glycoRNA signals (Figures 3C,D and S3A). Similarly, the application of Endo Hf, which causes glycosidic cleavage of the di-N-acetylchitobiose moiety on high-mannose structures,^37^ and Endo F2, which preferentially cleaves biantennary and high mannose structures,^38^ also had no discernible effect on the glycoRNA signal. These results stand in contrast to reports made by Flynn et al. and Ma et al., which suggested a partial or robust loss of signals of ManNAz-probed HeLa RNA following treatment with these endoglycosidases.^10,19^

Moreover, ManNAz-RNA, GlcNAz-RNA, and GalNAz-RNA signals remained unchanged after treatment with N-acetylglucosaminidase S (cleaves β-N-acetylglucosaminyl residues),^39^ α-N-acetylgalactosaminidase (cleaves terminal α linked N-acetylgalactosamine residues),^40^ or O-glycosidase (targets core 1 and core O-glycans).^41^ Interestingly, only α-2-3,6,8,9 neuraminidase A/sialidase treatment, which is selective for cleaving sialic acid residues from glycans,^41,42^ resulted in substantial elimination of ManNAz-RNA, GlcNAz-RNA, and GalNAz-RNA signals. This result raises questions when considering that Ac_4_GalNAz is shown not to convert to sialic acid.^43^

To ensure that the observed resistance to glycosidases was not due to the presence of DBCO-biotin, we modified the procedure sequence by clicking DBCO-biotin to ManNAz-RNA after glycosidase treatments, followed by sample purification using spin columns (Figure S3B). The results of this approach matched the initial results, with a significant decrease in signal only for sialidase, while the signals for endoglycosidases (PNGase, Endo Hf, F2, O-glycosidase) and other exoglycosidases (β-N-acetylglucosaminidase S and β-N-acetylglucosaminidase S) remained unchanged. This consistency reinforces that the enzyme resistance pattern observed in glycoRNA is not an artifact of the biotinylation process.

To further validate the activity of the glycosidases, we performed SDS-PAGE and Strep-IR blotting analysis on azido-sialic acid N-glycan modified fetuin (Figure S3C). Initial steps included desialylation and N-glycan enzymatic labeling of fetuin with CMP-azido-sialic acid and ST6GAL1.^44^ The significant reduction of the N-glycan signal in fetuin after treatment with glycosidases such as PNGase, Endo F2, and sialidase confirmed the activity of these enzymes and their capability to cleave N-glycoprotein signals. Therefore, the lack of impact of PNGase and Endo F2 on glycoRNA signals is not due to enzyme inactivity but rather from the presence of distinct glycan structures.

### Exploring Nonenzymatic Glycosylation of RNA

Intrigued by the resistance to enzymatic cleavage we investigated the possibility of nonenzymatic labeling reactions of RNA. Previous studies have reported that HexNAz, in their per-O-acetylated form (e.g., Ac_4_ManNAz, Ac_4_GlcNAz, and Ac_4_GalNAz), induce nonenzymatic cysteine S-glycosylation in various intracellular proteins, suggesting a potential analogous mechanism with RNA.^45,46^ Moreover, the documented side reactions between cyclooctyne in DBCO and cysteine residues or the termini of peptides or proteins further prompted us to explore nonenzymatic labeling possibilities in RNA.^47,48^

For our investigation, we treated nonprobed RNA derived from HeLa cells, as well as synthetic RNA with an excess of Ac_4_HexNAz under various conditions (Figure 4A). Following the removal of unbound Ac_4_HexNAz via a spin column, the purified RNA was conjugated to DBCO-biotin to facilitate detection through Strep-IR blotting. We observed an unexpected Strep-IR signal that indicates nonenzymatic labeling of the total nonprobed RNA after treatment with 10 mM of Ac_4_ManNAz, Ac_4_GlcNAz, and Ac_4_GalNAz at 37 °C for 24 h (Figures 4B and S4A). Of note, cellular RNA labeling was performed at 100 μM, at which no signal was detected with in vitro labeling (Figure S4B). We also noted a negligible difference in the Strep-IR signal between incubations with 2 and 10 mM Ac_4_ManNAz, suggesting a possible saturation effect or capacity limitation of the RNA to interact with the sugar probe (Figure S4B). RNase cocktail (A and T1) treatment removes both total RNA and biotinylated-RNA signals entirely which confirms the specificity of labeling (Figure S4B). This nonenzymatic labeling was not limited to RNA derived from culture of mammalian cell lines—we also observed consistent formation of the nonenzymatic Ac_4_ManNAz labeled nucleic acids across yeast tRNA, 18-mer synthetic RNA, and 19-mer synthetic DNA (Figure S4C,D). We found that for all the case of yeast tRNA and synthetic RNA and DNA, the SYBR gold and Strep-IR signals overlap at the same molecular weight area and that Strep-IR signal can only be observed only in lanes where RNA was treated both with the sugar probe and clicked with DBCO-biotin (Figure S4D), showing the covalent nature of this sugar-RNA molecule.

**Figure 4:**
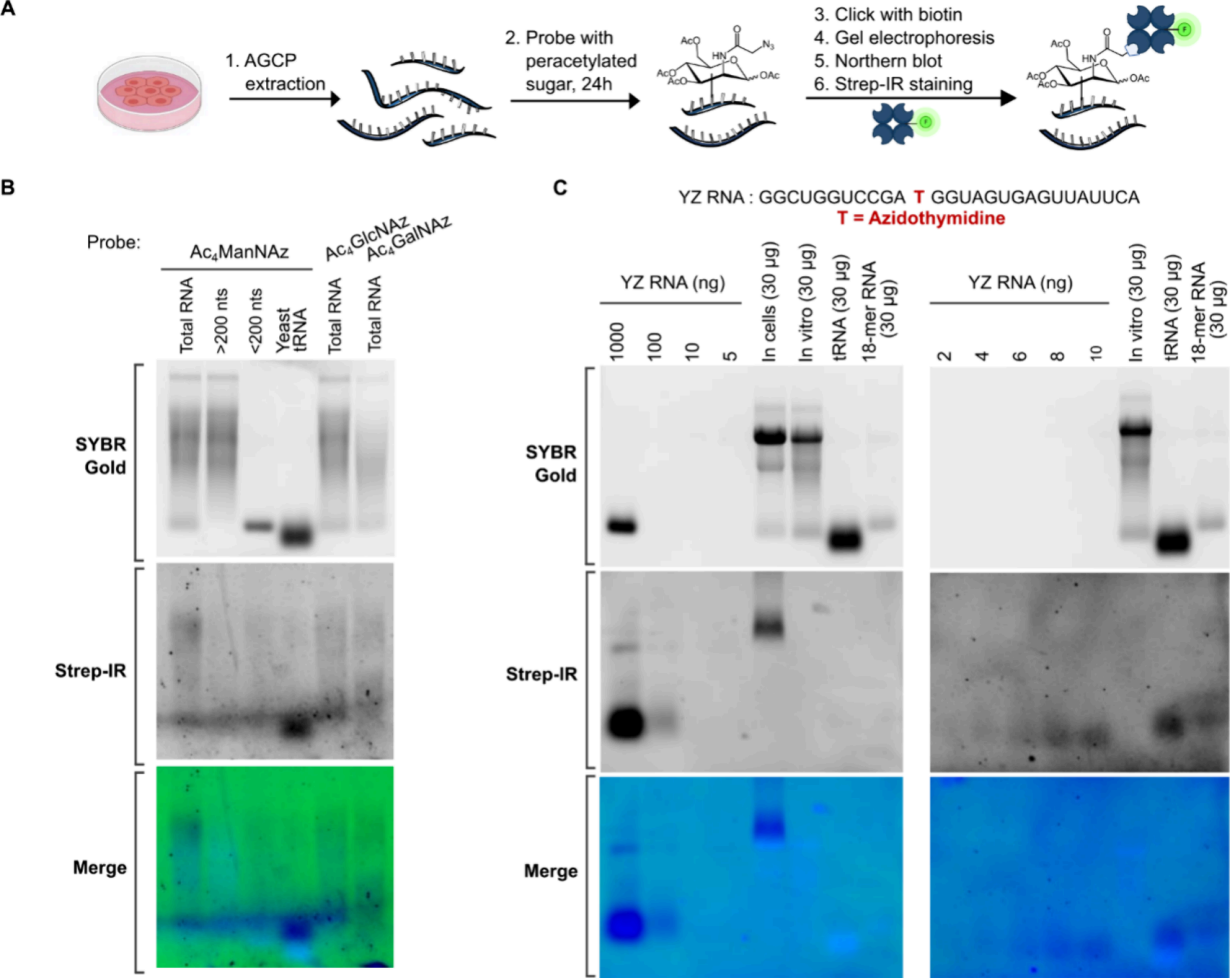
Protocol and results of in vitro reaction between RNA and per-O-acetylated sugar probes. (A) Schematic figure of the nonenzymatic labeling protocol using per-O-acetylated sugar probes on RNA. We first extracted total RNA from unprobed HeLa cells using the AGCP method, followed by ProtK and DNase treatments for cleanup. This isolated RNA was then incubated with an excess of Ac_4_ManNAz, purified via a spin column, and clicked with DBCO-biotin for visualization via Northern blot. (B) Gel electrophoresis (SYBR Gold) and Strep-IR blotting results of in vitro labeling of 50 μg of various RNA types (total RNA, large RNA (>200 nts), small RNA (<200 nts) or yeast tRNA) with either Ac_4_ManNAz, Ac_4_GlcNAz, or Ac_4_GalNAz, as indicated for each lane. Treatments were performed with 10 mM of the respective per-O-acetylated sugar at 37 °C for 24 h using 1× PBS buffer pH 7.4 as solvent. (C) Comparative analysis of synthetic azide-modified RNA (YZ RNA) ranging from 2 to 1000 ng and RNA samples treated with Ac_4_ManNAz both in cell and in vitro, including HeLa total RNA, yeast tRNA, and 18-mer synthetic RNA. Visualization was achieved through agarose gel electrophoresis (SYBR Gold) and Strep-IR blotting to demonstrate the efficiency of nonenzymatic labeling.

To discern the biological relevance of nonenzymatic RNA labeling reactions, we performed a comparative analysis to measure the extent of ManNAz-RNA formation both within cells and in vitro. Using synthetic azide-modified RNA (YZ RNA) as a benchmark, we could analytically gauge the degree of azido sugar incorporation (Figure 4C). The comparative analysis, visualized through agarose gel electrophoresis and Strep-IR blotting, revealed that the in vitro generation of ManNAz-RNA was substantially lower than the ManNAz-RNA levels observed within cells. This trend was consistent across different RNA types, with in vitro reactions showing only a fraction of the signal intensities seen in-cell. Given the significant discrepancies in signal intensities between in vitro and in vivo labeling, we conclude that chemical labeling has a negligible effect compared to enzymatic labeling in living cells.

### Enriched ManNAz-, GlcNAz-, and GalNAz-RNA Transcripts and Comparative Analysis with Initial Report

In order to identify the enriched small RNAs associated with the three probes, Ac_4_ManNAz, Ac_4_GlcNAz, and Ac_4_GalNAz, we employed high throughput small RNA sequencing. Given that glycosylation is expected to interfere with reverse-transcription during library preparation we identify those enriched small RNAs by virtue of their relative loss of sequencing depth as compared to controls. This approach provided us with an extensive data set, facilitating a comprehensive identification of putative glycoRNA transcripts that were commonly found among the three metabolic probes, as well as those unique to each probe. To prepare the RNA samples for sequencing, we isolated small RNA from probed HeLa cells treated with sugar probes, creating samples in biological triplicates. These samples had then subjected them to two different treatments: a control and a click sample preparation (Figure S5A). For the click samples, we conjugated the RNA with DBCO-biotin, while the control samples were left untreated. Subsequent enrichment of the samples used a streptavidin pulldown method, followed by rigorous washing and the release of the pulled RNA for library preparation. This approach enabled us to account for potential nonspecific enriched RNA hits due to streptavidin beads-pulldown bias.

Gel electrophoresis analysis using a Bioanalyzer and Northern blotting analysis of the triplicate control and click samples for each probe confirmed the presence of RNA and biotinylated glycoRNA solely in the click samples, validating successful enrichment and extraction of biotinylated sugar-labeled RNA off the beads (Figure S5B,C).

Subsequently, we used these RNA samples to generate a cDNA library using Bioo Scientific NEXTflex Small RNA-Seq Kit for sequencing on an Illumina NextSeq 2000. Following sequencing, the reads were deduplicated using the tally algorithm^49^ to exclude PCR duplicates and the library size of the samples indicated that GlcNAz-RNA control samples had the lowest library read (approximately 300,000 reads), yet no low-quality RNA-seq libraries were detected (Figure S6A). The sequences’ unique molecular identifiers (UMIs) and adapters were automatically trimmed using the reaper algorithm and reads that were less than 15nt after trimming were excluded from further analyses, given that they are too short to map meaningfully. The sequence lengths of all samples were plotted, revealing peaks at 21 nts and 70 nts, which aligns with the expected lengths of small RNAs (Figure S6B).

To facilitate precise mapping of small RNA, we established an annotation workflow. The sequences were cross-referenced against a database of human noncoding RNAs and filtered to select the best matches, employing a heuristic classification strategy in conjunction with a precedence annotation system (see 1.9 in SI). This strategy favors well-annotated, shorter RNA sequences but allows alignment to longer sequences when necessary. Differential gene expression analysis to identify depleted small RNAs was performed utilizing the DESeq2 package^50^ and rigorous quality assessment of the samples was conducted using PCA test which corroborated a high concordance among the biological replicates with no significant outliers (Figure S6C). Analysis of family classes across all control and click groups revealed a generally similar pattern, though control libraries exhibited more rRNA-mapping and less tRNA mapping reads than the click libraries (Figure S6D).

To isolate transcripts specifically enriched in ManNAz-, GlcNAz-, and GalNAz-RNA, we adopted stringent parameters, multiple tests corrected (Benjamini and Hochberg) *P*-Value of less than 0.05 for statistical significance^51^ and a log2­(ratio) greater than 0.5. The annotated transcripts of ManNAz, GlcNAz-, and GalNAz-RNA were visualized by volcano and MA plots to give the overall view of the transcripts with large magnitude of change (log2­(ratio)) and high mean counts (Figure 5A–F).

**Figure 5:**
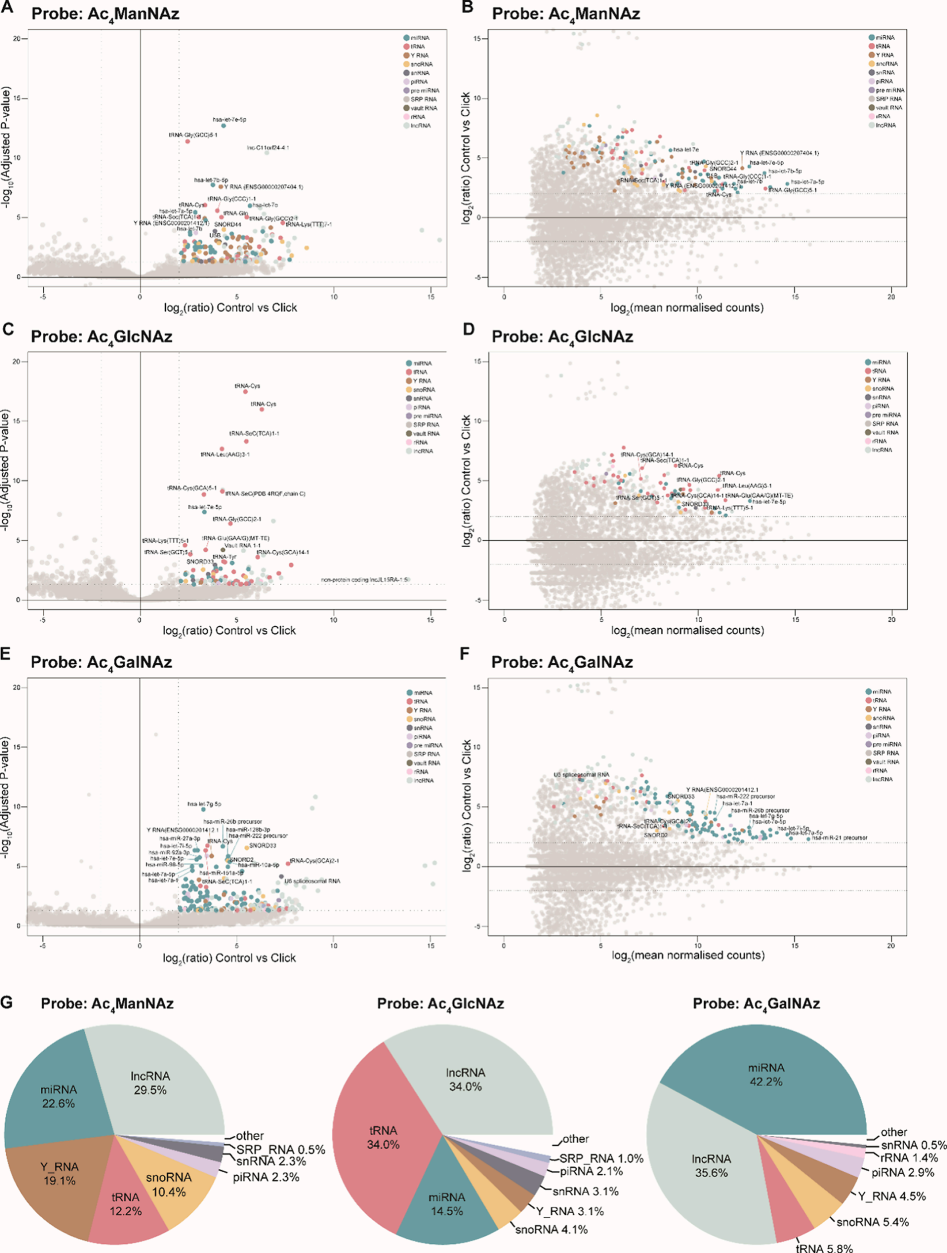
High throughput sequencing results of ManNAz-, GlcNAz-, and GalNAz-RNA. (A) Volcano plot analysis of ManNAz-RNA identified from HeLa small RNA. (B) MA plot analysis of ManNAz-RNA identified from HeLa small RNA. (C) Volcano plot analysis of GlcNAz-RNA identified from HeLa small RNA. (D) MA plot analysis of GlcNAz-RNA identified from HeLa small RNA. (E) Volcano plot analysis of GalNAz-RNA identified from HeLa small RNA. (F) MA plot analysis of GalNAz-RNA identified from HeLa small RNA. (G) Pie charts comparing the distribution of enriched libraries of each indicated sugar the between Control Click samples with *p*_adj_ value <0.05 and log 2­(ratio) > 0.5.

We identified unique sets of enriched transcripts associated with each sugar probe: ManNAz-RNA enriched transcripts were characterized by a significant prevalence of lncRNA (28.3%), miRNA (22.3%), YRNA (18.3%), and tRNA (12.2%) (Figure 5A,B,G). We defined 180 enriched transcripts with the two most statistically significant hits identified as hsa let-7e-5p and tRNA-Gly (GCC) 5-1 (Figure S7A). GlcNAz-RNA was dominated by tRNA (35.5%) and had a notable lncRNA (30%) and miRNA abundance (18.2%), leading to a total of 110 hit transcripts (Figure 5C,D,G). The top two hits in this category were HPN antisense RNA 1 and tRNA-Cys (Figure S7B). Contrastingly, GalNAz-RNA enrichment was primarily characterized by a high miRNA (42.4%) and lncRNA content (35.1%), followed by a smaller proportion of tRNA (6.3%) and snoRNA (5.4%) (Figure 5E–G). GalNAz-RNA has the highest number hit transcripts with a total of 208 hit transcripts with the top 20 listed in Figure S7C.

Having revealed commonalities and unique elements across the Ac_4_ManNAz, Ac_4_GlcNAz, and Ac_4_GalNAz enriched RNA populations, we identified a set of 26 transcripts that were consistently enriched among all three sugars (Figure 6A). This shared pool comprised of tRNAs, miRNAs, lncRNAs, YRNAs, and snoRNAs. The three most significant hits among this shared pool were tRNA-Cys, hsa-let-7e-5p, and tRNA-Sec (TCA) 1-1 (Figure 6B).

**Figure 6:**
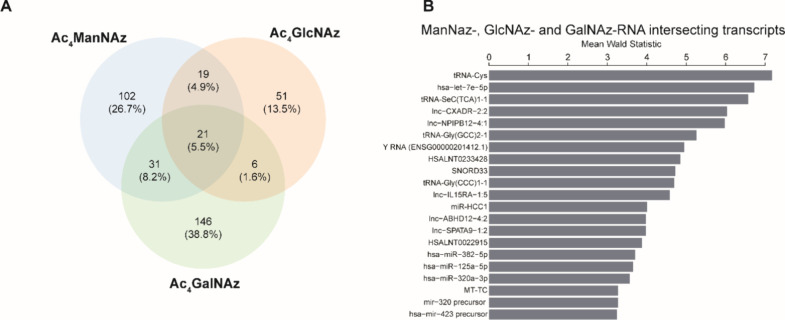
Comparative analysis of transcript overlaps and abundance among different glycoRNA from HeLa cells. (A) Venn diagram showing transcript overlap among ManNAz-, GlcNAz-, or GalNAz-RNA from HeLa cells. (B) List of all 21 intersecting transcripts between ManNAz-, GlcNAz-, or GalNAz-RNA ordered by decreasing Mean Wald Statistic values.

The shared pool of transcripts enriched across ManNAz-, GlcNAz-, and GalNAz-RNA presents possibilities regarding the mechanisms of RNA glycosylation. Selective enrichment of certain transcripts that intersected consistently across the three sugars seems to suggest a degree of enzymatic selectivity, or possible nucleophilicity of the amino acid side-chain on aminoacyl-tRNAs.

Our comparative analysis highlights that tRNAs and snoRNAs are the most likely candidates for bona fide carbohydrate functionalized RNAs. Interestingly, these RNA classes are known for their extensive post-transcriptional modifications even with monosaccharides. For instance, tRNA has been documented to be hypermodified with mannosyl- and galactosyl-queuosine.^11–13^ Nevertheless, lingering concerns remain, such as why we were unable to detect some of the statistically significant glycoRNA transcripts, such as Y5 and U1, highlighted in Flynn et al. and Ma et al.’s reports.^10,19^ These discrepancies may stem from differences in experimental procedures, methodologies, or the dynamic nature of RNA glycosylation, influenced by cellular context, location, and varying physiological states. Furthermore, the low stoichiometry and scarcity of this sugar-modified RNA, indicate a potential for significant variation at the single-cell level,^52^ adding to the complexity and challenges of glycoRNA transcriptome mapping.

## Discussion

In this study, we pursued a broader understanding of the presence and extent of RNA glycosylation by utilizing and expanded set of metabolic chemical reporters in their per-O-acetylated forms (Ac_4_ManNAz, Ac_4_GlcNAz, Ac_4_GalNAz, and Ac_4_FucAz). The enzymatic labeling enabled purification, visualization, and subsequently advanced sequencing technologies. We replicated earlier findings on glycoRNA detection using the Ac_4_ManNAz probe and successfully extended our study with probes Ac_4_GlcNAz and Ac_4_GalNAz. The results consistently showed signals in small RNAs (<200 bases) only and rigorous ProtK treatments confirmed minimal glycoprotein contamination. Even though the signal was completely removed after RNase treatment, a potential coprecipitation of glycosylated molecules due to purification with silica-based spin column cannot be ruled out.^53^ Differences in ManNAz-RNA signals among different cell types were noted, and the Ac_4_FucAz probe did not yield any glycoRNA signals, suggesting potential sugar incompatibilities in RNA glycosylation.

Our study also revealed a highly resistant bond in ManNAz-, GlcNAz-, and GalNAz-RNA, to enzymatic cleavage by most endoglycosidases such as PNGase, Endo Hf, and Endo F2 but not sialidase. The discovery deviates from previous glycoRNA reports^10,19^ and prompted hypotheses about potentially novel RNA-sugar interactions, which could be akin to nonenzymatically labeled species. Therefore, in vitro experiments, using a direct, enzyme-independent reaction between per-O-acetylated azido sugars and RNA, demonstrated that nonenzymatic labeling only has a minor effect on glycoRNA detection. The modification of both total RNA isolated from HeLa cells and synthetic oligonucleotides only occurred at high concentrations of the reporter and did not result in the characteristic shift of the signal to higher apparent molecular weights after gel electrophoresis. These results confirm that the glycoRNA, isolated from probed cells, stems from metabolic incorporation of the reporters and not from unwanted nonenzymatic pathways.

We sought to comprehend the cellular context of RNA glycosylation by conducting an in-depth investigation of its transcriptomic landscape. To mitigate the bias from nonspecific RNA binding to streptavidin, we employed a stringent pulldown-release methodology coupled with a novel annotation workflow. This method, coupled with our data processing pipeline using a novel annotation workflow, facilitated the identification of unique enriched transcripts associated with each Ac_4_ManNAz, Ac_4_GlcNAz and Ac_4_GalNAz sugar probe. Importantly, we discovered that certain transcripts were selectively enriched across all three probes. This is reinforced by the observation that labeling with all three probes occurs exclusively in small RNA. Still, we interpret these results with caution, given the diversity of glycosylated RNA transcripts and incomplete overlap of statistically significant transcripts. This emphasizes the need for in-depth exploration of glycoRNA, specifically measuring modified individual RNA copy numbers, best achieved using robust methods like single-cell RNA-Seq and direct sequencing without reverse-transcription. Our sequencing results indicate that tRNAs are the most promising candidates for genuine carbohydrate-functionalization, deserving thorough biochemical characterization. Furthermore, tRNAs are among the most highly enriched extracellular RNA species, an observation not at odds with the assertion that glycoRNAs are localized on the cell surface.^10,54,55^

Collectively, this work provides a broader perspective on the metabolic labeling of glycoRNA showing the successful incorporation of an expanded repertoire of metabolic chemical reporters. It is important, however, to acknowledge inherent limitations. Our study is constrained by the nature of metabolic chemical reporters in their per-O-acetylated forms, including its metabolic conversion, limited uptake and uneven cellular distribution. Overall, our study aims to guide scientific inquiry to the limitations, challenges, and uncertainties of the emerging field of RNA glycosylation. This emphasizes the necessity for scrutiny in such nascent research with the overarching objective of further elucidating the nature of glycoRNA and reshaping our understanding of RNA- and glycobiology.

## Methods

### Mammalian Cell Culture

HeLa, HEK293T, and PANC1 cells were cultured in DMEM + GlutaMAX media (Gibco) supplemented with 10% v/v fetal bovine serum (FBS). SKRC52, WA-C3CD5+, MOLM13, Hel, and Jurkat cells were cultured in RPM1–1640 media with glutamine (Gibco) supplemented with 10% v/v FBS. SKBR3 cells were cultured in McCoy’s 5A + GlutaMAX media (Gibco) supplemented with 10% v/v FBS. All cells were grown in T75 or T175 CELLSTAR Standard Culture Flasks with standard screw cap red at 37 °C and 5% CO_2_. Cells were maintained at >85% viability and were passaged every 3 days (or as needed). All cells were authenticated using short tandem repeat (STR) profiling and tested negative for mycoplasma contamination. All adherent cells were dissociated using TrypLE (recombinant protein). Cells were seeded for experiments at >80% viability. Biological triplicates were generated from different passage numbers of different batches of cells.

### Metabolic Chemical Reporters

Stocks of N-azidoacetyl-mannosamine-tetra acetylated (Ac_4_ManNAz, Sigma-Aldrich), N-azidoacetyl-glucosamine-tetra acetylated (Ac_4_GlcNAz, Carbosynth), N-azidoacetyl-galactosamine-tetra acetylated (Ac_4_GalNAz) and 6-azidofucose-tetra acetylated (Ac_4_FucAz), were prepared in sterile dimethyl sulfoxide (DMSO) in a concentration of 500 mM. For labeling experiments, 3 × 10^6^ HeLa cells in 10 mL DMEM + GlutaMAX media were plated on cell culture dishes and incubated for 24 h. The cells were then incubated with the respective probe at a final concentration of 100 μM at 37 °C and 5% CO_2_ for 24 h.

### Cell Lysis and Initial RNA Extraction by AGPC

To the cells on 150 mM Nunc EasYDish (Thermo Fischer Scientific) was directly added 3 mL of Qiazol reagent (Qiagen). Cells were scraped directly on the plate without the use of dissociation agents. After homogenization in Qiazol by pipetting, the solution was stored at −80 °C and RNA extraction was performed within 30 days of storage. For RNA extraction out of Qiazol solution, first phase separation was initiated by adding 200 μL of chloroform per 1 mL of Qiazol solution. The suspension was rigorously mixed and subsequently centrifuged at 12,000 × *g* for 15 min at 4 °C. The aqueous phase was carefully transferred to a new microcentrifuge tube, mixed with 500 μL of isopropanol, vortexed to mix, and kept at r.t. for 10 min. The mixture was spun at 12,000 × *g* for 10 min. The supernatant was carefully removed, and the RNA pellet was washed with 1000 μL of 75% EtOH followed by centrifugation at 7500 × *g* for 5 min. After EtOH was removed, the RNA pellet was air-dried for 30–60 min and then dissolved in nuclease-free water. RNA concentration was determined with a NanoDrop.

### Proteinase K (ProtK) Treatment

To ensure that RNA is free of glycoprotein, RNA was subjected to protein digestion by adding 1 μg of Proteinase K (Prot K, Thermo Fisher Scientific) per 25 μg of total RNA. The final concentration of ProtK was 10 μg/mL and the concentration of RNA was 250 ng/μL. ProtK treatment was performed in nuclease-free water at 37 °C for 45 min. Subsequently, the RNA sample was further purified using a Zymo-Spin IIICG column according to the manufacturer’s protocol.

### Separation of Small and Large RNA

The crude sample after ProtK treatment was purified using a Zymo-Spin IIICG column (approximately 100–300 μg of RNA per column) according to the manufacturer’s instructions. The total sample was mixed with 1 × volume of RNA Binding Buffer (Zymo research) and 1 × volume of EtOH. The resulting mixture (∼83 ng/μL) was passed through a Zymo-Spin IIICG column in four equal portions, retaining large RNA (>200 nts) within the column. The filtrate, containing small RNAs (<200 nts), was mixed with an equal volume of EtOH and mixed by pipetting up and down. The resulting mixture was passed through an equivalent number of Zymo-Spin IIICG columns as used in the initial step, requiring the sample be added to the column in approximately eight equal portions to retain small RNA within the column. Subsequently, columns containing either large or small RNA fractions were treated with 400 μL RNA Prep Buffer, followed by sequential washes with 700 μL and then 400 μL RNA Washing Buffer (Zymo Research). RNA was finally eluted using 100–200 μL of RNase-free water and concentration measured with a NanoDrop.

### DNase Treatment of RNA Samples

Lyophilized RNA was dissolved in nuclease-free water to a concentration of 222 ng/μL. 10× TURBO DNase buffer (Thermo Fisher Scientific) was added to the RNA solution to give a final RNA concentration of 200 ng/μL. For every 10 μg of RNA, 1 μL of TURBO DNase (2 U/μL, final concentration 0.04 U/ μL) was added and the resulting mixture was incubated at 37 °C for 1 h. Following DNase treatment, RNA was purified using a Zymo-Spin ICG according to the manufacturer’s protocol. Approximately 50 ng of RNA was applied to each column and RNA was eluted in nuclease free water (80 μL per column).

## Supplementary Material









## Data Availability

The raw sequencing data for this study is deposited in the European Nucleotide Archive (ENA) under accession PRJEB64789.

## Notes

General synthetic methods; preparation of metabolic probes; click conjugation conditions; agarose gel electrophoresis and Northern blot analysis; proteinase K (Figure S1), DNase and RNase treatment conditions; glycosidase treatment on glycoRNA and sialylated Fetuin (Figure S3); in vitro labeling with sugar probes (Figure S4); enrichment and release data (Figure S2); and sequencing experiments (Figures S5–S7) (PDF)
Enriched ManNAz-, GlcNAz-, and GalNAz-RNA (XLSX)
Intersecting hits ManNAz-, GlcNAz-, and GalNAz-RNA (PDF)
Score counts (XLS)
