# Supplementary material for: An Expanded View of RNA Modification with Carbohydrate-Based Metabolic Probes
Source: JACS Au. 2025 May 5;5(5):2309–20. doi: 10.1021/jacsau.5c00249 (PMC12117391; doi:10.1021/jacsau.5c00249)
Supplement: Supplementary file 1 [file au5c00249_si_001.pdf]

Supporting Information

An Expanded View of RNA Modification with Carbohydrate-Based Metabolic Probes

Madoka E. Hazemi[1], Michael B. Geeson[1†], Felix M. Müller[1†], Sigitas Mikutis[1], Anton J. Enright[2*], Gonçalo J. L. Bernardes[1,3,4*]

[1]Yusuf Hamied Department of Chemistry, University of Cambridge, Lensfield Road, Cambridge, CB2 1EW, United Kingdom.

[2]Department of Pathology, University of Cambridge, Tennis Court Road, Cambridge, CB2 1QP, United Kingdom.

[3]GiMM - Gulbenkian Institute for Molecular Medicine, Avenida Prof. Egas Moniz, 1649-028 Lisboa, Portugal.

[4]Translational Chemical Biology Group, Spanish National Cancer Research Centre (CNIO), Madrid 28029, Spain.

[†] These authors contributed equally.

*Corresponding author. Email: aje39@cam.ac.uk; gb453@cam.ac.uk.

Table of Contents

1. Materials and Methods 3
   1.1 General synthetic methods 3
   1.2 Preparation of metabolic probes 3
   1.3 Click conjugation of glycoRNA 4
   1.4 Agarose gel electrophoresis and northern blot analysis 4
   1.5 Sensitivity of glycoRNA to enzymatic treatment 5
   1.6 Glycosidase treatment on azido-sialylated Fetuin 7
   1.7 In vitro non-enzymatic RNA labelling with sugar probes 8
   1.8 Enrichment and release of labelled RNA by biotin pulldown 9
   1.9 Next-generation sequencing (NGS) experiments 10
2. Supplementary Figures 14
3. References 23

1. Materials and Methods

1.1 General synthetic methods

All chemicals and dry solvents were purchased from commercial suppliers and used without further purification. All non-aqueous reactions were performed in oven-dried glassware under inert atmosphere unless otherwise stated. Reaction vessels were heated using thermostatically controlled DrySyn blocks filled with sand. Water used experimentally was deionised and prepared on site. Flash column chromatography was performed using silica gel technical grade with a particle size of 60 µm (Merck). Analytical thin layer chromatography was performed using silica gel 60 µm particle size (Merck) F254 and visualised by UV (254 nm), by staining with a $KMnO_4$ or $(NH_4)_4Ce(SO_4)_4$ solution. NMR spectra were recorded on a 400 MHz AVIII HD Smart Probe Spectrometer or a 600 MHz Avance 600 BBI Spectrometer. HPLC analysis and purification were carried out on Thermo Fisher Scientific Ultimate 3000 HPLC system, using NUCLEOSIL 100-5 C18 semi-preparative column. Mobile phase: A) 0.1% formic acid in H_2O and B) 0.1% formic acid in MeCN. Flow rate = 3 mL/min.

1.2 Preparation of metabolic probes

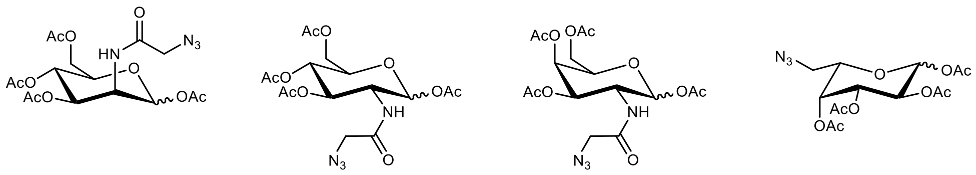

Ac_4ManNAz Ac_4GlcNAz Ac_4GalNAz Ac_4FucAz

Ac_4ManNAz and Ac_4GlcNAz were purchased from commercial suppliers. Ac_4GalNAz and Ac_4FucAz were prepared according to published methods and the analytical data was in accordance with the reported literature.[1,2]

1.3 Click conjugation of glycoRNA

SPAAC

To RNA (50 µg) in nuclease-free water (60 µL) was added dye-free GLB-II (75 µL) followed by DBCO-biotin in DMSO (15 µL, final concentration 500 µM) to give a total volume of 150 µL. The reaction mixture was incubated at 55°C for 10 min and quenched by addition of 2× volumes of RNA Binding Buffer (300 µL, Zymo Research), followed by addition of 3× volumes of 100% EtOH (450 µL). The mixture was purified over a Zymo-Spin IC column (Zymo research) following the manufacturer's protocol. RNA was then eluted in 50 µL of nuclease-free water and the concentration is measured with a NanoDrop™. Control reactions were performed by following the procedure above with pure DMSO in place of a solution of DBCO-biotin in DMSO.

CuAAC

To RNA (50 µg) in nuclease-free water (128 µL) was added $CuSO_4$ (4 µL, final concentration 100 µM), THPTA (4 µL, final concentration 500 µM), sodium ascorbate (4 µL, final concentration 1 mM), sodium phosphate buffer pH 7.5 (50 µL, final concentration 50 mM) and alkyne-biotin or alkyne-fluor 488 (10 µL, final concentration 500 µM) to give a final volume of 200 µL. The reaction mixture was incubated at 37°C for 15 min and quenched by addition of 2× volumes of RNA Binding Buffer (400 µL, Zymo Research), followed by addition of 3× volumes of 100% EtOH (600 µL). The mixture was purified over a Zymo-Spin IC column (Zymo research) following the manufacturer's protocol. RNA was then eluted in 50 µL of nuclease-free water and the concentration is measured with a NanoDrop™. Control reactions were performed by repeating the above protocol with pure water in place of the aqueous solution of $CuSO_4$.

1.4 Agarose gel electrophoresis and northern blot analysis

To make an 7 × 7 cm^2 of denaturing agarose gel, 300 mg of agarose was dissolved in 27 mL pure water using a microwave, then 3 mL of 10× NorthernMax™ Denaturing Gel Buffer (Thermo Fisher Scientific) was added and the gel was let set at r.t. for at least 1 h. RNA sample is diluted in up to 15 µL water and mixed with 15 µL of FEB (95% formamide, 10 mM EDTA, pH 8.2) before charging. When applicable, 5 µL of 100-3000 bp DNA ladder was added to the gel (GeneRuler 100 bp Plus DNA Ladder, Thermo Fisher Scientific).

For visualisation of the RNA, to each sample and ladder was added 1× SYBR gold (Thermo Fisher Scientific) in DMSO. When direct addition of 1× SYBR gold to samples was not suitable, gels were stained after electrophoresis with a mixture of 1× SYBR Gold in 1× MOPS NorthernMax™ buffer (Thermo Fisher Scientific) for 10 min. Electrophoresis was performed at 100 V for 25 min in 1× MOPS Northern Max buffer. RNA in gel was detected using Chemidoc (Bio-Rad) imaging system using the SYBR Gold channel. For transferring the RNA onto a nitrocellulose membrane, a 3 cm stack of 10 × 10 cm^2 tissues, 8 pieces of 9 × 9 cm^2 filter paper, and 1 piece of 9 × 9 cm^2 of 0.45 mm nitrocellulose membrane (NC, Cambridge Bioscience) were used. The transfer was carried out for 1.5–2 h for a 0.6 cm thick, 8 × 8 cm^2 surface area denaturing agarose gel. After transfer, RNA was cross-linked to the NC membranes using UV-C light (0.18 J/cm^2). NC membranes were then blocked with 15 mL Intercept PBS blocking buffer (LiCor Biosciences) for 45 min at room temperature. After blocking, NC membranes were stained with 15 mL of 1× Streptavidin-IR 8000 in Intercept PBS blocking buffer at r.t. for 30 min. Excess Streptavidin-IR 8000 was removed from the membranes by three washes with 0.1% Tween-20 (Sigma) in 1× PBS at r.t. for 5 min each. NC membranes were briefly rinsed with 1× PBS to remove the Tween-20 before imaging with detection of the signal using Chemidoc (Bio-Rad) imaging

system using the IR-800 channel. When appropriate, gel images signal was quantified with the Image Lab 6.1 software (Bio-Rad) in the IR-800 channel.

1.5 Sensitivity of glycoRNA to enzymatic treatment

Total RNA labelled with biotin described in sections Cell lysis and initial RNA extraction by AGPC and Click conjugation of RNA were subjected to systematic treatments with enzymes:

First ProtK treatment

RNA was subjected to protein digestion by adding 1 µg of ProtK (Thermo Fisher Scientific) per 25 µg of total RNA. The final concentration of RNA was 250 ng/µL and the final concentration of ProtK is 10 U/µL. ProtK treatment was performed in pure water at 37ºC for 45 min. Following treatment with ProtK, RNA was directly purified using a Zymo-Spin ICG column (Zymo research) according to the manufacture's protocol. Approximately 50 ng of RNA was applied to each column and RNA was eluted in nuclease-free water (50 µL per column). The concentration of purified RNA was calculated using a NanoDrop^{TM} and then click-conjugated with DBCO-biotin as detailed in Section 1.3. A total of 15 µg of the RNA in 10 µL of water was used for gel electrophoresis and northern blot analysis as detailed in Section 1.4.

Second ProtK treatment

To ensure the total removal of glycoprotein, 20 µg of RNA obtained after ProtK treatment and click conjugation (Methods section, Proteinase K (ProtK) Treatment) are subjected to another round of protein digestion by adding 1 µg of ProtK per 25 µg of total RNA with the final RNA concentration of 250 ng/µL. Treatment was performed in nuclease-free water at 37°C for 45 min. Following treatment with ProtK, RNA was directly purified using a Zymo-Spin ICG column (Zymo research) according to the manufacture's protocol. RNA was eluted in nuclease-free water (15 µL per column) and the concentration of purified RNA was calculated using a NanoDrop^{TM}. A total of 15 µg of the RNA in 10 µL of water was used for gel electrophoresis and northern blot analysis as detailed in Section 1.4.

DNase treatment

To 20 µg of total RNA after ProtK treatment and click conjugation, as described in the methods section, was added 10 × TURBO DNase buffer (Thermo Fisher Scientific) to give a final RNA concentration of 200 ng/µL. For every 15 µg of RNA, 1.5 µL of TURBO DNase (2 U/ µL, Thermo Fisher Scientific) was added and the resulting mixture incubated at 37°C for 1 h. Following DNase, RNA was directly purified using a Zymo-Spin ICG column (Zymo research) according to the manufacture's protocol. RNA was eluted in nuclease-free water (15 µL per column) and the concentration of purified RNA was calculated using a NanoDrop^{TM}. A total of 15 µg of the RNA in 10 µL of water was used for gel electrophoresis and northern blot analysis as detailed in Section 1.4.

RNase treatment

To 20 µg of total RNA after ProtK treatment and click conjugation as described in the methods section Proteinase K (ProtK) Treatment was added nuclease-free water to a concentration of 200 ng/µL. 10× RNase buffer (200 mM Tris-HCl (pH 8.0), 1 M KCl and 1 mM MgCl) was added to the RNA solution to give a final RNA concentration of 200 ng/µL.

For every 15 µg of RNA, 1.5 µL of RNase cocktail (5 U/µL RNase A and 200 U/µL RNase T1 (final concentration: 0.1 U/ µL RNase A and 4 U/ µL RNase T1), Thermo Fisher Scientific) was added and the resulting mixture incubated at 37 ºC for 1.5 h. Following treatment with RNase, RNA was directly purified using a Zymo-Spin ICG column (Zymo research) according to the manufacturer's protocol. RNA was eluted in nuclease-free water (15 µL per column) and the concentration of purified RNA was

calculated using a NanoDrop™. A total of 15 µg of the RNA in 10 µL of water was used for gel electrophoresis and northern blot analysis as detailed in Section 1.4.

Analysis of enzyme treated glycoRNA

15 µg of the purified RNA samples after enzyme treatment in 10 µL nuclease-free water were added to 15 µL of FEB (Formamide Extracting Buffer, 95% Formamide, 18 mM EDTA) and then analysed by gel electrophoresis and northern blot analysis as detailed in Section 1.4.

Different Conditions of ProtK Treatment

Total RNA from 2 biological replicates was click-conjugated with DBCO-biotin as detailed in Section 1.3 and then treated with ProtK in three different conditions:

- Condition A: To 30 µg of the click-conjugated total RNA was added ProtK (final concentration 10 ug/mL) and nuclease-free water to a final RNA concentration of 250 ng/µL. The resulting mixture was incubated at 37°C for 45 min.
- Condition B: To 30 µg of the click-conjugated total RNA was added ProtK (final concentration: 250 ug/mL), 7.5 × ProtK Buffer (75 mM Tris HCl (pH 7.5), 7.5 mM EDTA, 750 mM NaCl), 10 M Urea (final concentration: 4 M), and nuclease-free water to a final RNA concentration of 250 ng/µL. The resulting mixture was incubated at 50 ºC for 1 hr.
- Condition C: To 30 µg of the click-conjugated total RNA was added ProtK (final concentration: 500 ug/mL), 7.5 × ProtK Buffer (75 mM Tris HCl (pH 7.5), 7.5 mM EDTA, 750 mM NaCl), 10 M Urea (final concentration: 4 M), and nuclease-free water to a final RNA concentration of 250 ng/µL. The resulting mixture was incubated at 50 ºC for 1 hr.

Following the treatment with ProtK, RNA was directly purified by biotin-pulldown method and extracted out of streptavidin beads as detailed in Section 1.8 to get pure RNA in formamide for gel electrophoresis and northern blot analysis as detailed in Section 1.4.

ProtK treatment on biotinylated protein

To 0.5 µg of the biotinylated protein control (C1-biotin, final concentration 0.01 µg/µL) was added ProtK (final concentration 10 µg/mL) in RNase free water and the mixture was incubated at 37°C for 45 min. For SDS PAGE gel electrophoresis, protein concentrations were normalised across all samples and ~0.2 µg of samples were mixed with NuPAGE™ 4 x LDS buffer (NP0007; Invitrogen). Afterwards, samples were loaded on NuPAGE™ 4-12% Bis-Tris precast gels (Invitrogen) and a molecular weight marker (ab116027; Abcam or SeeBlue™ Plus2; Thermo Fisher Scientific) was loaded alongside to determine the size of individual proteins. Gel electrophoresis was performed using 1 x NuPAGE™ MES SDS running buffer (Thermo Fisher Scientific) in a XCell Mini-Cell (Thermo Fisher Scientific) for 35 min at 200 V. SDS-Page gel was used for protein transfer for blotting analysis or was stained with InstantBlue™ Coomassie Stain (Abcam) to image separated proteins using ChemiDoc (Bio-Rad). To transfer proteins separated by SDS PAGE gel electrophoresis from the gel to PVDF membranes (iBlot™ 2 Transfer Stacks, PVDF, mini; Invitrogen), protein transfer was performed using the iBlot 2 Gel Transfer Device (IB21001; Thermo Fisher Scientific) using the P0 method for 7 min transfer. The membranes containing the transferred proteins were blocked immediately by incubation in 15 mL Intercept PBS blocking buffer (LiCor Biosciences) at r.t. for 45 min. After blocking, NC membranes were stained with 15 mL of 1× Streptavidin-IR 8000 (LiCor Biosciences) in Intercept PBS blocking buffer (LiCor Biosciences) at r.t. for 30 min. Excess Streptavidin-IR 8000 was removed from the membranes by three washes with 0.1% Tween-20 (Sigma) in 1× PBS at r.t. for 5 min each. NC membranes were briefly rinsed with 1× PBS to remove the Tween-20 before imaging with detection of the signal using Chemidoc (Bio-Rad) imaging system using the IR-800 channel.

Endo- and exo-glycosidases treatment

All digestions were performed on 30 µg of total RNA from two biological replicates that was treated with ProtK (Methods section) and was click-conjugated with DBCO-biotin as detailed in Section 1.3. All digestions were performed at 37°C for 1 h.

- PNGase F: 30 µg RNA was subjected to 3 µL of PNGase F (500 U/µL, NEB), in 1 x GlycoBuffer 2 (50 mM sodium phosphate (pH 7.5), NEB) and nuclease-free water to give a final RNA concentration of 1000 ng/µL.
- Endo Hf: 30 µg RNA was subjected to 3 µL of Endo Hf (1000 U/µL, NEB), in 1x GlycoBuffer 3 (50 mM sodium acetate (pH 6.0), NEB) and nuclease-free water to give a final RNA concentration of 1000 ng/µL.
- Endo F2: 30 µg RNA was subjected to 3 µL of Endo F2 (8U/µL, NEB) in 1x GlycoBuffer 4 (50 mM sodium acetate (pH 4.5), NEB) and nuclease-free water to give a final RNA concentration of 1000 ng/µL.
- α-2-3,6,8,9 Neuraminidase A (Sialidase): 30 µg RNA was subjected to 3 µL of α-2-3,6,8,9 Neuraminidase A (50 U/µL, NEB) in 1x GlycoBuffer 2 (50 mM sodium phosphate (pH 7.5), NEB) and nuclease-free water to give a final RNA concentration of 1000 ng/µL.
- β-N-Acetylglucosaminidase S: 30 µg RNA was subjected to 3 µL of β-N-Acetylglucosaminidase S (4 U/µL, NEB) in 1x GlycoBuffer 1 (50 mM sodium acetate (pH 5.5), 5 mM $CaCl_2$, NEB) and nuclease-free water to give a final RNA concentration of 1000 ng/µL.
- α-N-Acetylgalactosaminidase: 30 µg RNA was subjected to 3 µL of α-N-Acetylgalactosaminidase (20000 U/µL, NEB) in 1x GlycoBuffer 1 (50 mM sodium acetate (pH 5.5), NEB) and nuclease-free water to give a final RNA concentration of 1000 ng/µL.
- O-Glycosidase: 30 µg RNA was subjected to 3 µL of O-Glycosidase (40000 U/µL, NEB), in 1x GlycoBuffer 2 (50 mM sodium phosphate (pH 7.5), NEB) and nuclease-free water to give a final RNA concentration of 1000 ng/µL.

1.6 Glycosidase treatment on azido-sialylated Fetuin

Preparation of desialylated Fetuin

Mix 10 µg Fetuin (ThermoFischer Scientific, final concentration 1 µg/µL) with 0.1 µg of rcpNeuraminidase (R&D systems, final concentration 0.01 µg/µL) in Assay Buffer pH 7.5 (final concentration 25 mM HEPES, 150 mM NaCl). Incubate for 20 minutes at room temperature. Inactivate rcpNeuraminidase by heating at 90°C for 5 minutes.

CMP-azido-sialic acid labelling (N-glycan) to desialylated Fetuin

Combine 10 µg of desialylated Fetuin from the previous step with 2 µg of rhST6GAL1 (R&D systems, final concentration 0.04 µg/µL) and 1 nmol of CMP-Azido-Sialic Acid (final concentration 0.02 mM) in Assay Buffer pH 7.5 (final concentration 25 mM HEPES, 150 mM NaCl) supplemented with 10 mM $MnCl_2$ to a final volume of 50 µL. Incubate at 37°C for 30 minutes.

For negative controls: Prepare controls by following the reaction setup but omit rhST6GAL1.

Glycosidase Treatment

Enzymatic Digestion: Subject 1 µg of Fetuin-azido-sialic acid to 1 µL of specific glycosidase treatments (PNGase F, Endo Hf, Endo F2, α-2-3,6,8,9 Neuraminidase A, β-N-Acetylglucosaminidase S, α-N-Acetylgalactosaminidase, O-Glycosidase) in 1x of their respective GlycoBuffers to a final protein concentration of 0.0625 µg/µL. Incubate at 37°C for 1 hour.

Biotinylation

To each sample of Fetuin-azido sialic acid (final concentration 0.04 µg/µL), 1 mM DBCO-biotin (final concentration 0.125 mM) in RNase free water. Incubate at 37°C for 2 hours.

SDS PAGE gel electrophoresis

For SDS PAGE gel electrophoresis, each protein was first passed through Zeba™ MicroSpin Desalting Columns 7K MWCO 75 µL (Thermo Fisher Scientific). Protein concentrations were then normalised across all samples and ~0.5 µg of each sample was mixed with NuPAGE™ 4 x LDS buffer (NP0007; Invitrogen). Afterwards, samples were loaded on NuPAGE™ 4-12% Bis-Tris precast gels (Invitrogen) and a molecular weight marker (ab116027; Abcam or SeeBlue™ Plus2; Thermo Fisher Scientific) was loaded alongside to determine the size of individual proteins. Gel electrophoresis was performed using 1 x NuPAGE™ MES SDS running buffer (Thermo Fisher Scientific) in a XCell Mini-Cell (Thermo Fisher Scientific) for 35 min at 200 V. SDS-Page gel was used for protein transfer for blotting analysis or was stained with InstantBlue™ Coomassie Stain (Abcam) to image separated proteins using ChemiDoc (Bio-Rad).

Protein transfer and blotting analysis

To transfer proteins separated by SDS PAGE gel electrophoresis from the gel to PVDF membranes (iBlot™ 2 Transfer Stacks, PVDF, mini; Invitrogen), protein transfer was performed using the iBlot 2 Gel Transfer Device (IB21001; Thermo Fisher Scientific) using the P0 method for 7 min transfer. The membranes containing the transferred proteins were blocked immediately by incubation in 15 mL Intercept PBS blocking buffer (LiCor Biosciences) for 45 min at room temperature. After blocking, NC membranes were stained with 15 mL of 1× Streptavidin-IR 8000 in Intercept PBS blocking buffer at r.t. for 30 min. Excess Streptavidin-IR 8000 was removed from the membranes by three washes with 0.1% Tween-20 (Sigma) in 1× PBS at r.t. for 5 min each. NC membranes were briefly rinsed with 1× PBS to remove the Tween-20 before imaging with detection of the signal using Chemidoc (Bio-Rad) imaging system using the IR-800 channel.

1.7 In vitro non-enzymatic RNA labelling with sugar probes

Total RNA in-vitro non-enzymatic labelling

To 50 µg of total RNA (500 ng/µL) from non-probed HeLa cells, extracted by AGCP method and after treatment with ProtK and DNase (Method section), was added 500 mM per-O-acetylated sugar azide reporters (final concentration 0-10 mM) in either 1x PBS buffer pH 7.4 + 2% DMSO or DMEM + GlutaMAX™ media (Gibco) + 2% DMSO to a final volume of 100 µL at 37°C for 2 h, 5 h, or 24 h. The resulting RNA mixture was quenched by addition of 2× volumes of RNA Binding Buffer (200 µL, Zymo Research), followed by addition of 3× volumes of 100% EtOH (300 µL). The mixture was purified over a Zymo-Spin IC column (Zymo research) following the manufacturer's protocol and the RNA was eluted in 50 µL of nuclease-free water. The purified RNA was then subjected to DBCO-biotin (Section1.3) and treated with RNase if applicable (Section 1.5). The concentration of purified RNA was calculated using a NanoDrop™ and a total of 30 or 50 µg of the RNA was used for gel electrophoresis and northern blot analysis as detailed in Section 1.4.

Yeast tRNA, 18-mer synthetic RNA and 19-mer synthetic DNA in-vitro non-enzymatic labelling

To 50 µg of yeast tRNA (ThermoFischer Scientific, final concentration 500 ng/µL) or 18-mer synthetic RNA (Sequence: CACAUCAGCACAACUACG, IDT DNA, final concentration 500 ng/µL), or 19-mer synthetic DNA (Sequence: GTGCGTCGATGAAGAACGC, final concentration 500 ng/µL) was added 500 mM per-O-acetylated sugar azide reporters (final concentration 10 mM) in 1x PBS buffer pH 7.4 to a final volume of 100 µL at 37°C for 2 h, 5 h, or 24 h. The resulting RNA mixture was quenched by addition of 2× volumes of RNA Binding Buffer (200 µL, Zymo Research), followed by addition of 4.5× volumes of 100% EtOH (450 µL). The mixture was purified over a Zymo-Spin IC column (Zymo research) following the manufacturer's protocol and the RNA was eluted in 50 µL of nuclease-free water. The purified RNA was then subjected to DBCO-biotin (Section 1.3). The concentration of purified RNA was calculated using a NanoDrop^{TM} and a total of 30 µg of the RNA was used for gel electrophoresis and northern blot analysis as detailed in Section 1.4.

1.8 Enrichment and release of labelled RNA by biotin pulldown

RNA was extracted, treated with ProtK, treated with DNase, separated into large and small RNA fractions, and biotinylated according to the methods section. Enrichment of biotinylated RNA was achieved using streptavidin-coated magnetic beads (MyOne C1 Streptavidin beads, Thermo Fisher Scientific). The protocol below was adapted for enrichment for quantities down to as low as 5–10 µg of purified small RNA. All washing steps were performed by inverting the microcentrifuge tube containing magnetic beads five times, briefly centrifuging, magnetizing for 2 min, and removal of the solution with a pipette. Care was taken to avoid drying of the beads. A list of buffers used to enrich and wash biotinylated RNA is provided in Table S1.

Table S1. Buffers used for enrichment and washing of biotinylated RNA on beads.

Buffer	Composition
**B&W Buffer**	5 mM Tris HCl, pH 7.5, 0.5 mM EDTA, 1 M NaCl
**Solution A**	0.1 M NaOH, 0.05 M NaCl
**Solution B**	0.1 M NaOH
**High-Salt Wash Buffer (HSWB)**	100 mM Tris HCl, pH 7.4, 10 mM EDTA, 1 M NaCl, 0.05% Tween-20
**Biotin Wash Buffer (BWB)**	10 mM Tris HCl, pH 7.5, 1 mM EDTA, 100 mM NaCl, 0.05% Tween-20
**Saline-Sodium Citrate (SSC) Buffer (20×)**	3 M NaCl, 300 mM sodium citrate, pH 7
**chIRP Buffer**	2 × SSC Buffer, 0.5% SDS
**NT2 Buffer**	50 mM Tris HCl, pH 7.5, 150 mM NaCl, 1 mM
**High Urea Washing Buffer (HUWB)**	6M Urea, 0.01M EDTA
**Gel Loading Buffer-II (GLB-II)**	Invitrogen Catalogue Number AM8546G
**Dye-free GLB-II (FEB)**	95% Formamide, 18 mM EDTA

Beads preparation

Streptavidin-coated magnetic beads (10 µL, 0.1 mg, binding capacity of ~50 pmol biotinylated ss-oligonucleotide) were washed with 1× B&W buffer (1 × 1 mL), solution A (2 × 1 mL), and solution B (1 × 1 mL). Then beads were blocked with 50 ng/mL glycogen in HSWB (750 µL) for 1 h at 25°C then washed with HSWB (1 × 1 mL).

RNA binding

Biotinylated RNA (25 µg) was diluted in 750 µL BWB and mixed with the beads for 2 h at 4°C on a rotating shaker.

RNA washing

After binding, the beads underwent washing according to one of the following protocols:

- Standard washing protocol:

  Beads were washed with washing buffers: ChIRP Buffer (2 × 0.5 mL), HSWB (2 × 0.5 mL), NT2 Buffer (2 × 0.5 mL) and nuclease-free water (1 × 0.5 mL).

- Urea only protocol:

  Beads were washed with HUWB (2 × 0.5 mL) and nuclease-free water (1 × 0.5 mL),

- Standard washing + urea protocol:

  Beads first underwent washing step with washing buffers: ChIRP Buffer (2 × 0.5 mL), HSWB (2 × 0.5 mL), NT2 Buffer (2 × 0.5 mL). Followed by urea washing with: HUWB (2 × 0.5 mL) and nuclease-free water (1 × 0.5 mL).

RNA extraction

Beads were resuspended in formamide (10 µL), transferred to a PCR tube, and briefly centrifuged. The PCR tube was heated in a PCR thermocycler to 95°C for 2 min, with a lid temperature of 110°C. The PCR tube was magnetized for 2 min and the formamide containing release RNA was directly analysed (Section 1.5) or transferred to a fresh microcentrifuge tube containing nuclease-free water (190 µL). The resulting RNA solution was lyophilized overnight, and carefully redissolved in nuclease-free water.

1.9 Next-generation sequencing (NGS) experiments

Reverse transcription of released RNA and qPCR

30 µg of ProtK and DNase treated small derived from $Ac_4ManNAz$ in biological triplicates (Methods section) was subjected to DBCO-biotin for click samples and DMSO for control samples and purified by spin-column. (Section 1.3). Enrichment and release processes were then performed on this RNA material following the protocol of beads enrichment and release with standard washing, as detailed in Section 1.8. Following release, the RNA was redissolved in 18 µL of nuclease-free water.

A volume of 3 µL was subsequently set aside for northern blotting analysis (Section 1.4), and the remaining 15 µL were used for cDNA synthesis. For cDNA synthesis, to 15 µL of released RNA was added 4 µL of SuperScript™ VILO™ Master Mix (Thermo Fisher Scientific) and 1 µL water and the reaction mixture was incubated at 25°C for 10 min, then at 42°C for 60 min, and finished with 85°C for 5 min to terminate the reaction and to release the cDNA from the beads. Quantification of specific RNAs were measured using fast mode of Real-Time PCR System QuantStudio 3 (Applied Biosystems) and

Fast SYBR Green Master Mix (Applied Biosystems) according to the manufacturer's instructions. Primer sequences are listed in Table 2.

Table S2. List of primers.

Primer names	Primer Sequences (5′→3′)
**SNORD33 F**	ACTTCTCCCACTCACATTCG
**SNORD33 R**	GGGTGGCCTCAGATGGTAG
**SNORD44 (U44) F**	GAGCTAATTAAGACCTTCATGTTCA
**SNORD44 (U44) R**	AAATGCAGCCTGGATGATGA
**RNAY1 F**	GGCTGGTCCGAAGGTAGTGAG
**RNAY1 R**	GGGGGAAAGAGTAGAACAAGG
**U1 F**	CAGGGGAGATACCATGATCACGAAG
**U1 R**	CGCAGTCCCCCACTACCACAAAT

Sequencing samples preparation

ProtK and DNase treated small RNA, derived from each of the three probes, namely $Ac_4ManNAz$, $Ac_4GlcNAz$, and $Ac_4GalNAz$, were prepared in biological triplicates (Methods section). SPAAC was subsequently performed on these samples (Section 1.3) using DBCO-biotin for click samples and DMSO for control samples. This procedure yielded a final 32 µg of RNA materials per each sample, and 2 µg of each processed material were set aside as input samples. Enrichment and release processes were then performed on the remaining RNA material following the protocol of beads enrichment and release with standard washing, as detailed in Section 1.8.. Following release, the RNA was redissolved in 18 µL of nuclease-free water. A volume of 3 µL was subsequently set aside for northern blotting analysis, and the remaining 15 µL was used for sequencing library preparation.

Library preparation and small RNA sequencing

Library preparation was performed using Bioo Scientific NEXTflex® Small RNA-Seq Kit v3 and sequenced on an Illumina NextSeq 2000. Reads in FASTQ files are first deduplicated to exclude PCR duplicates using the tally algorithm and then UMIs and adapter sequences were removed automatically using the reaper algorithm. This leaves reads of varying length from 1 to 70nt. Reads less than 15nt after trimming are excluded from further analysis as they are too short to map meaningfully.

Iterative Classification Algorithm

Sequences are mapped against a FASTA file consisting of the RNA Central database of human non-coding RNAs. This is done using blastn with word_size set to 9 and a percentage identity threshold of 95% and forward strand searching only as follows:

```
blastn -query QUERYFILE -db rnacentral_human.fasta -outfmt "6 qseqid sseqid pident length mismatch gapopen qstart qend sstart send evalue bitscore qlen slen" -num_threads 2 -strand plus -word_size 9 -evalue 10 -perc_identity 95 -out QUERY.blasthits
```

An additional filter is then applied to the resulting hits obtained from BLAST. We demand that the length of the alignment of our small RNA against its match in the database be at least 80% of the length of the input sequence and that it have no more than 4 mismatches.

In many instances a small RNA will have still have alignments to multiple RNAcentral molecules with the same significance score. In these instances, we proceed through a heuristic classification strategy that takes into account the relative annotation qualities of different databases. For example, microRNAs and snoRNAs are smaller and better annotated so a significant match across the full length of a mature microRNA will preferentially be classified as a mature microRNA. In the case of a mature microRNA, it could feasibly match to mature microRNA (miRbase), precursor microRNA (miRbase) and the host transcript of the precursor such as a lncRNA (RFAM, LNCBASE). In the case of a 21nt molecule matching all these databases with the same score, we utilise a precedence annotation system where shorter better annotated matches are preferred over short matches against longer sequences with less confident annotations. This strategy achieves a balance between sensitivity and annotation accuracy. Sequences originating from poorly annotated classes of small RNAs can still find a match, but sequences from well characterised small RNA classes will preferentially match those first before matches in less well annotated small RNA families are explored. The precedence is set below:

**Database**	**Precedence Level**
MIRBASE	1
SNODB	2
SNOPY	3
5SRRNADB	4
GTRNADB	5
SRPDB	6
TMRNA_WEB	7
RFAM	8
LNCIPEDIA	9
LNCBOOK	10
LNCRNADB	11
LNCBASE	12
PIRBASE	13
GENECARDS	14
MALACARDS	15
REFSEQ	16
ENSEMBL	17

Once assigned to an RNA central family member based on these precedence, match and length constraints, we store that read as a member of that RNA central accession and keep the family and subfamily information from RNA central for future processing. The number of reads matching each RNA central accession from each sample are further stored in a count table which is then used with DESeq2 for normalisation and differential expression analysis.

Statistical Analysis

Small RNAs with a glycosylation are expected to fail the RT step and not produce duplexes which can generate sequencing reads. The mapped RNA counts table is normalised using DESeq2 and a negative-binomial wald test is used to identify mapped small RNAs where the number of reads has significantly reduced between control and test samples. Those small RNAs without significant glycosylation should produce similar levels between control and test samples. Hence we use both a $\log_2$ fold-change and a multiple testing corrected P-Value. Significant hits were deemed those with a $\log_2$ fold change ≤ -0.5 and an adjusted P-Value ≤ 0.05. In these cases the number of reads was quantitatively and significantly diminished between the test and control samples across multiple replicates.

2. Supplementary Figures

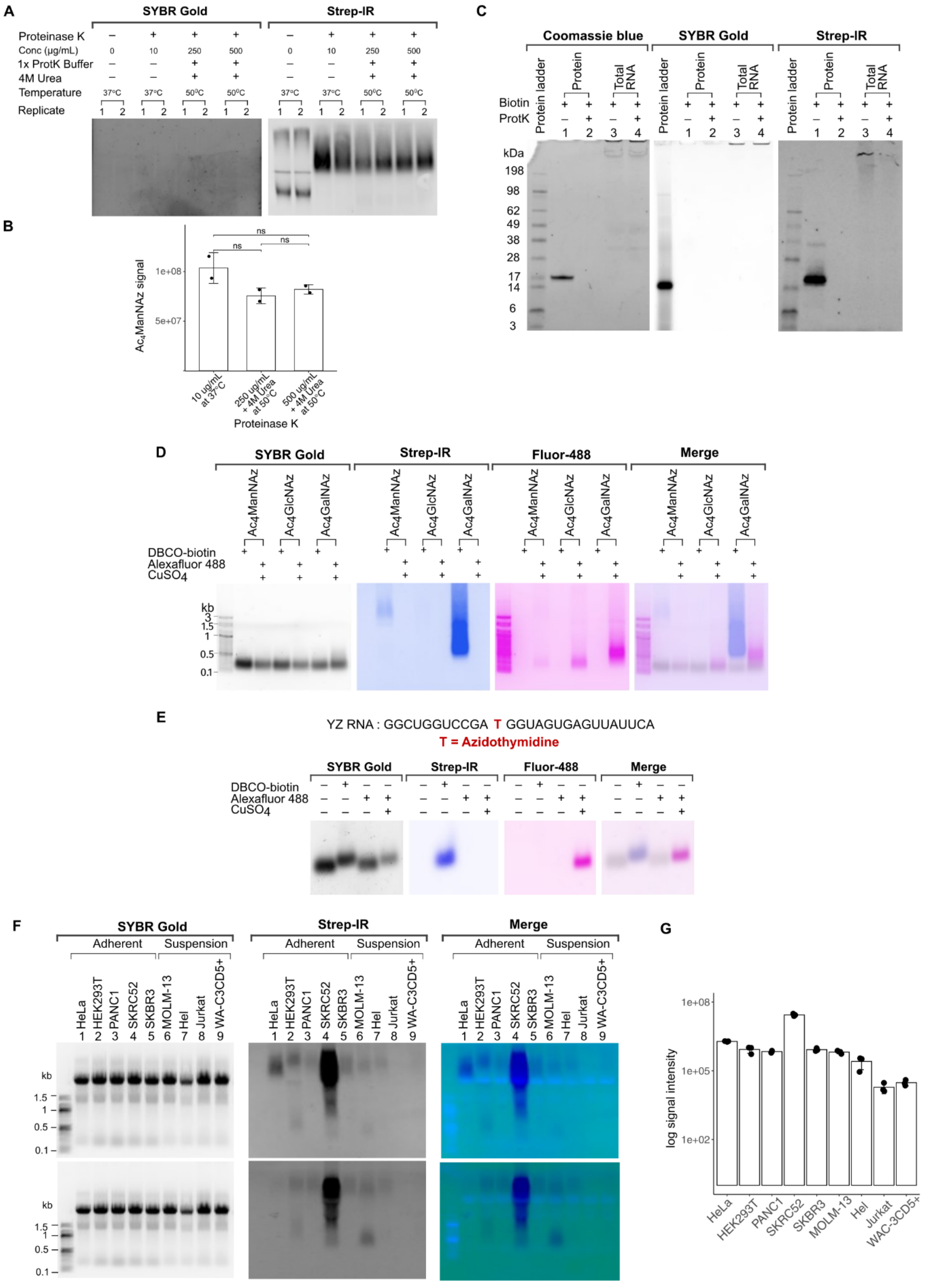

Fig. S1 Comprehensive analysis of ManNAz-RNA post-ProtK treatment and comparison across various conditions and cell types.

(A) Gel electrophoresis and Strep-IR blotting results of ManNAz-RNA from HeLa cells after in vitro treatment with ProtK with the indicated various conditions, including different ProtK concentrations (10 μg/mL or 250 μg/mL), temperatures (37°C or 55°C), and the presence of 4M urea. RNA purification after ProtK treatment for blotting was performed using the streptavidin-pulldown and release method. (B) Quantification of ManNAz-RNA signal post in vitro ProtK treatment under diverse conditions with error bars representing the standard deviation (SD) to illustrate the variability within the two biological replicates. Significance level ns indicates not significant (p-value > 0.05). (C) Western blotting (SDS-PAGE gel) of 0.2 μg of biotinylated protein and 10 μg of ManNAz-RNA from HeLa before and after in vitro treatment with 10 mg/ μL ProtK at 37°C for 1h. The blot was stained with Streptavidin-IR800 (Strep-IR) to visualise biotinylated protein or RNA. (D) Detection of ManNAz-small RNA from HeLa by clicking with DBCO-biotin followed with Strep-IR blot, in comparison with direct detection by clicking with alkyne-fluor 488. (E) Detection of synthetic azide RNA (YZ RNA) by clicking with DBCO-biotin followed with Strep-IR blot, compared to direct detection by clicking with alkyne-fluor 488. (F) Gel electrophoresis and Strep-IR blotting results of total RNA from various cancer cells probed with ManNAz including HeLa, HEK293T, PANC1, SKRC52, SKBR3, MOLM-13, Hel, Jurkat, WAC-3CD5+ grouped by their adherent or suspension cell type. (G) Quantification of ManNAz-RNA signal in various cancer cells in biological triplicates with error bars representing the standard deviation (SD) of the two biological replicates.

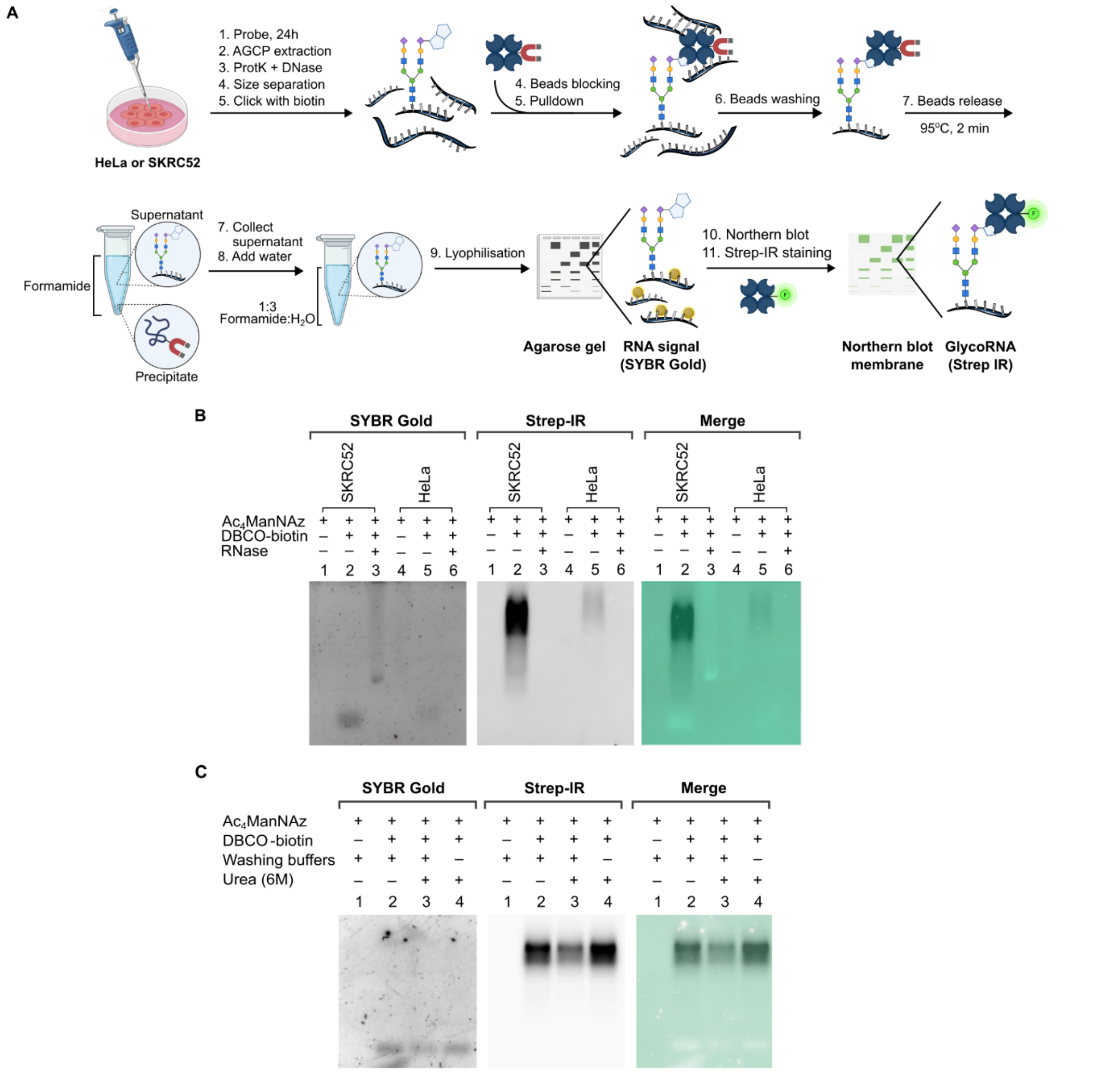

Fig. S2 Enrichment, release, and analysis of biotinylated ManNAz-small RNA from the beads

(A) Schematic representation of the enrichment and release protocol of enrichment and release of biotinylated ManNAz-small RNA from magnetic streptavidin beads. Biotinylated ManNAz-small RNA was prepared from SKRC52 or HeLa cells and bound to the magnetic streptavidin beads, which were previously blocked at 4°C for at least 2 hours. Non-specific RNA binding to streptavidin was exhaustively washed to remove impurity. The bound biotinylated RNA was then released by adding formamide and heating at 95°C for two minutes. The released RNA in formamide was collected by dilution with three times the volume of water, followed by lyophilisation, and finally elution with pure nuclease-free water to the preferred volume. The biotinylated RNA in the water was then visualised via agarose gel and SYBR Gold staining, followed by northern blotting and Strep-IR staining of the membrane after RNA transfer. (B) Gel electrophoresis and Strep-IR blotting results of ManNAz-small RNA from SKRC52 and HeLa cells after beads enrichment and release. The RNA on beads was washed with standard washing buffers before released, with treatments indicated for DBCO-biotin conjugation and RNase digestion pre-enrichment. (C) Gel electrophoresis and Strep-IR blotting results of ManNAz-small RNA from SKRC52 cells after beads enrichment and release. The RNA on beads was washed with either standard washing buffers and/or a 6M urea washing buffer to illustrate the impact of different washing condition on the purity and recovery of biotinylated RNA following release from the beads.

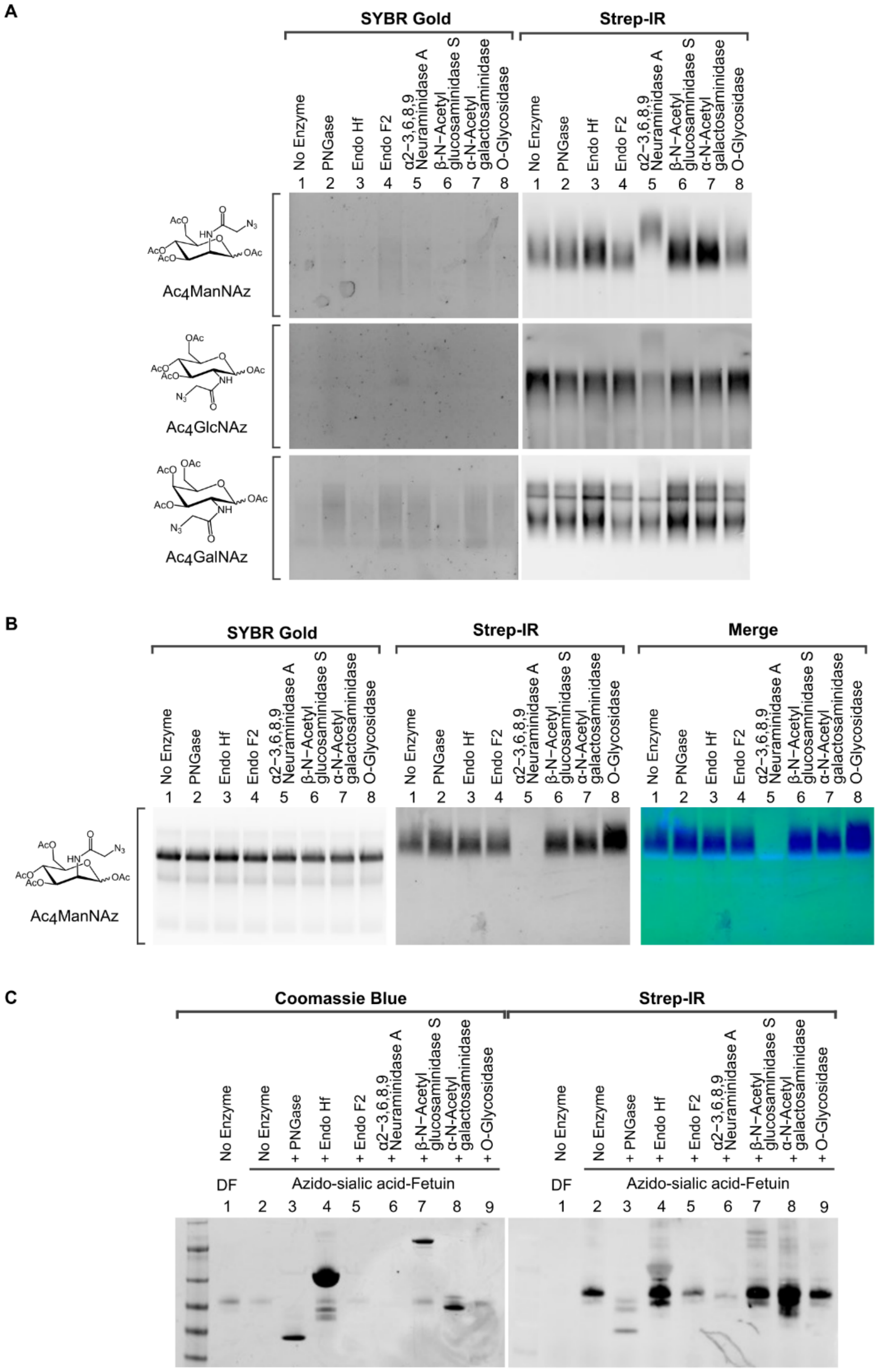

Fig. S3 Analysis of GlycoRNA and Glycoprotein modifications through glycosidases treatment

(A) Gel electrophoresis and Strep-IR blotting of ManNAz-, GlcNAz-, and GalNAz-total RNA from HeLa, biotinylated by clicking with DBCO-biotin, followed by enzymatic treatment with specified glycosidases at 37°C for 1 hour. The treated RNA was then purified via streptavidin pulldown enrichment and release method before analysis. (B) Gel electrophoresis and Strep-IR blotting of ManNAz-total RNA from HeLa treated with glycosidases followed by biotinylation through DBCO-biotin clicking. The treated RNA was then purified via spin column before analysis. (C) SDS-PAGE and Strep-IR blotting display of modified Fetuin. The protein underwent initial desialylation through Neuraminidase treatment. Subsequently, desialylated Fetuin was enzymatically labelled with CMP-azido-sialic acid facilitated by ST6GAL1 (lane 2), alongside a control without ST6GAL1 (lane 1). The azido-sialic acid-labelled Fetuin was then subjected to various glycosidases and conjugated with

DBCO-biotin (lanes 3-9). The processed samples were visualized via protein staining on SDS-PAGE (left panel) and Strep-IR detection on blotted membranes (right panel), illustrating the effects of glycosidase treatment on the biotinylated N-glycan protein.

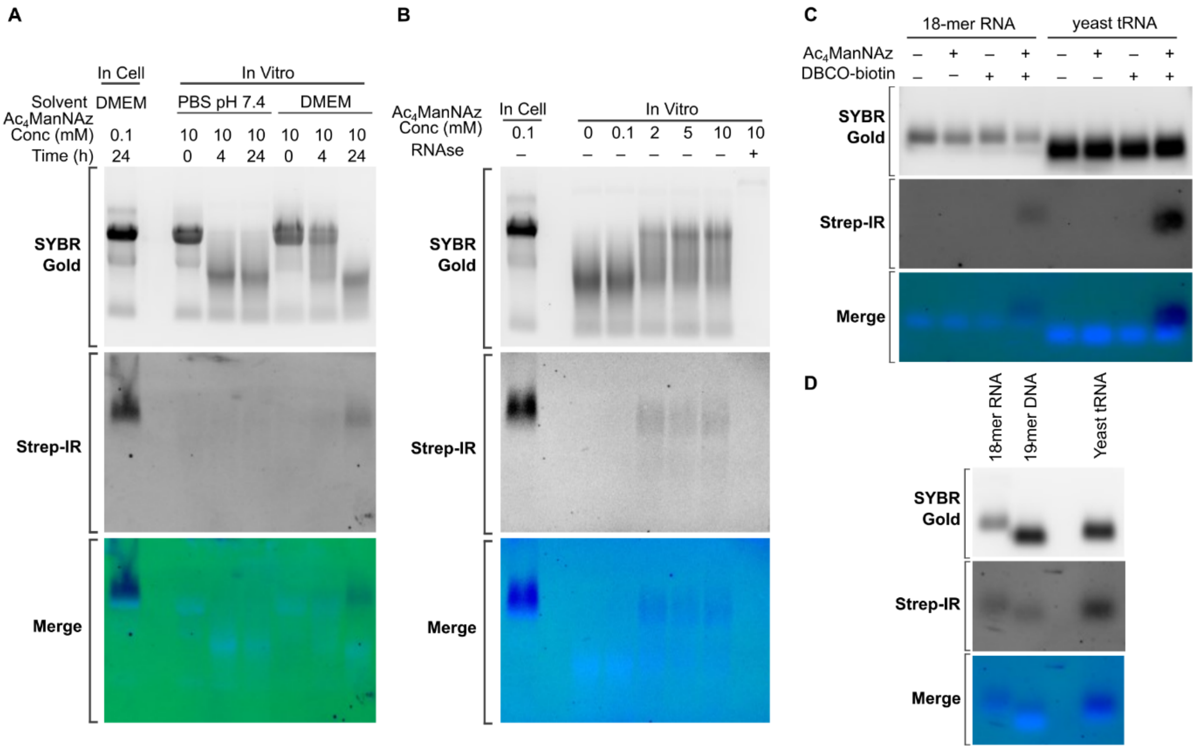

Fig. S4 Investigation of in vitro labeling of total RNA, yeast tRNA, synthetic RNA, and DNA with $Ac_4ManNAz$ under varying conditions

(A) Gel electrophoresis and Strep-IR blotting results of in vitro treatments of 50 µg non-probed total RNA with $Ac_4ManNAz$ under varying buffer conditions, as indicated for each lane. We conducted the treatment using 10 mM of $Ac_4ManNAz$ at 37°C across different time points. For comparison, we used the blotting of 10 µg $Ac_4ManNAz$ probed-RNA in HeLa cells (first lane) as a positive control. (B) Gel electrophoresis and Strep-IR blotting results of in vitro treatments of 50 µg non-probed total RNA with various concentrations of $Ac_4ManNAz$, as specified for each lane. The treatments were performed at 37°C for 24 h using DMEM as solvent. For comparison, we used the blotting of 10 µg $Ac_4ManNAz$ probed-RNA in HeLa cells (first lane) as a positive control. (C) Gel electrophoresis and Strep-IR blotting results of in vitro treatments of 50 µg 18-mer synthetic RNA or yeast tRNA with or without 10 mM $Ac_4ManNAz$, and subsequent clicking with DBCO-biotin or not, as indicated. The treatments were performed at 37°C for 24 h using 1x PBS pH 7.4 as solvent. The signals in Strep-IR indicated that non-enzymatic labelling occurs only in the presence of both $Ac_4ManNAz$ and DBCO-biotin. (D) Gel electrophoresis and Strep-IR blotting results of in vitro treatments of 50 µg 18-mer synthetic RNA, 19-mer synthetic DNA or yeast tRNA with 10 mM $Ac_4ManNAz$. The treatments were performed at 37°C for 24 hours using 1x PBS pH 7.4 as solvent and subsequent clicking with DBCO-biotin.

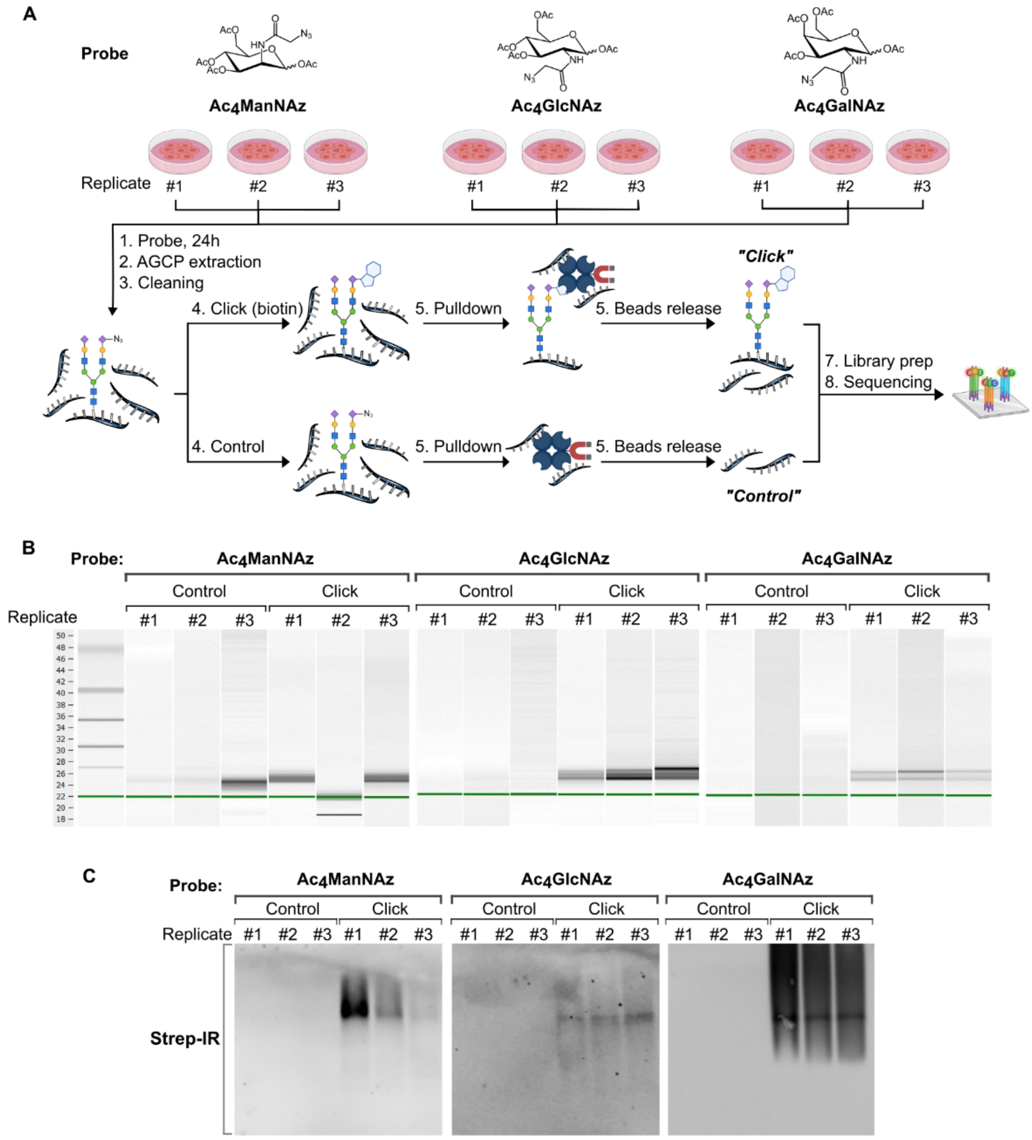

Fig. S5 Procedure and verification of ManNAz-, GlcNAz-, and GalNAz-RNA enrichment for sequencing analysis.

(A) Schematic of the method used to prepare the control and click samples for sequencing. After small RNA isolation and purification from cells, RNA was either conjugated DBCO-biotin for click samples or mixed with DMSO for control samples before then enriched using streptavidin-pulldown. The pulled RNA was then released from the beads for library preparation and Illumina-based small RNA sequencing. The experiment was carried out in biological triplicates. (B) Gel image from Bioanalyzer® analysis of ManNAz-, GlcNAz-, or GalNAz-RNA control and click samples after streptavidin-pulldown and release protocol. These RNA are used for library preparation using NETflex® kit. (C) Gel electrophoresis and Strep-IR blotting of ManNAz-, GlcNAz-, or GalNAz-RNA control and click samples after streptavidin pulldown and release protocol. The strep-IR signal only showed on click samples indicating successful targeted enrichment and release of biotinylated sugar-RNA.

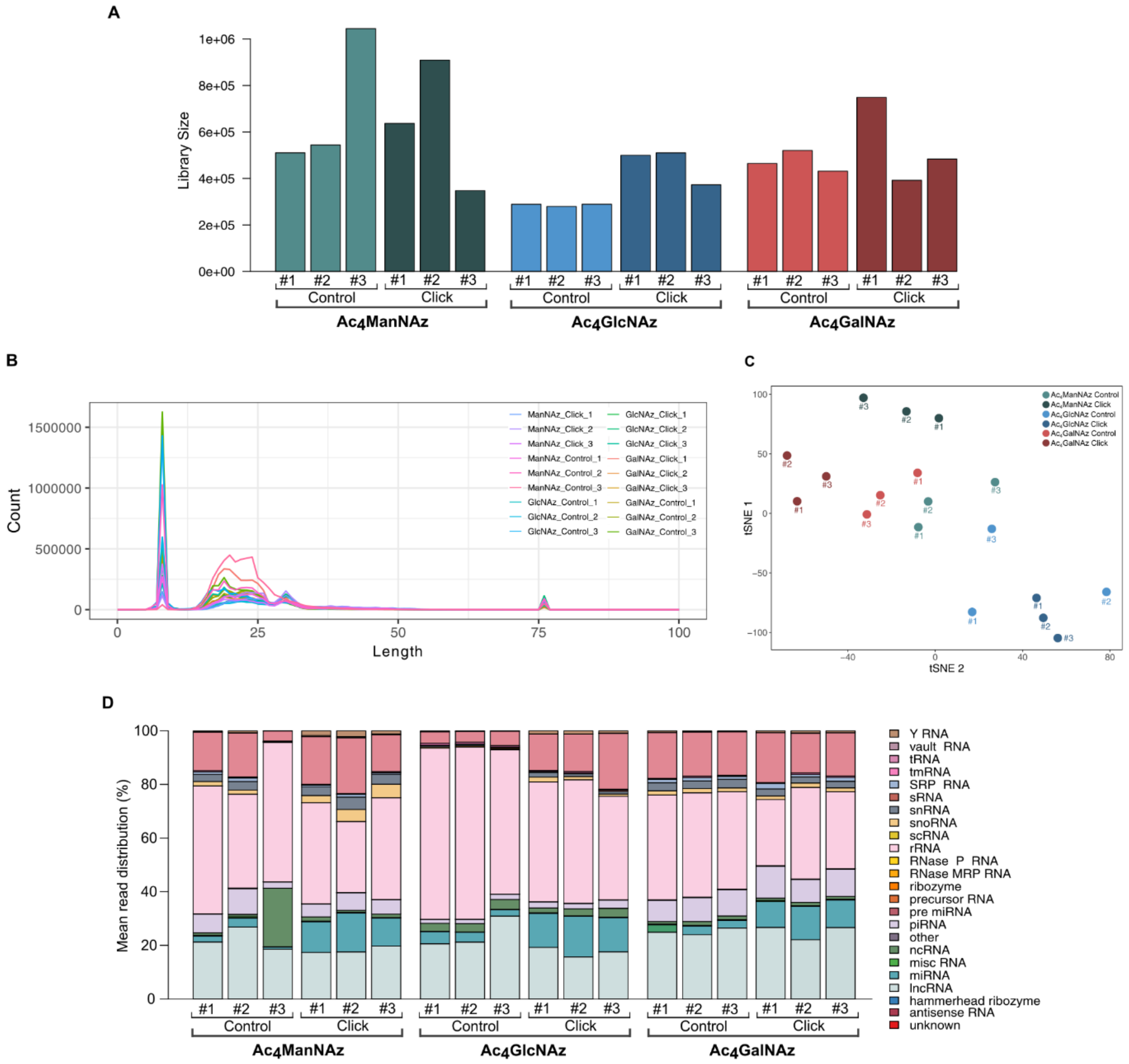

Fig. S6 Characterization and quality assessment of sequenced ManNAz, GlcNAz-, and GalNAz-RNA libraries.

(A) Comparison of library sizes of control and click samples from Ac_4ManNAz, Ac_4GlcNAz, and Ac_4GalNAz-enriched libraries. (B) Representation of read size distributions for ManNAz-, GlcNAz-, and GalNAz-RNA post-adaptor trimming. (C) tSNE analysis visualization of control and click samples from Ac_4ManNAz, Ac_4GlcNAz, or Ac_4GalNAz-enriched libraries. (D) Mean read distribution, segmented by RNA family type, following Bowtie alignment for the libraries.

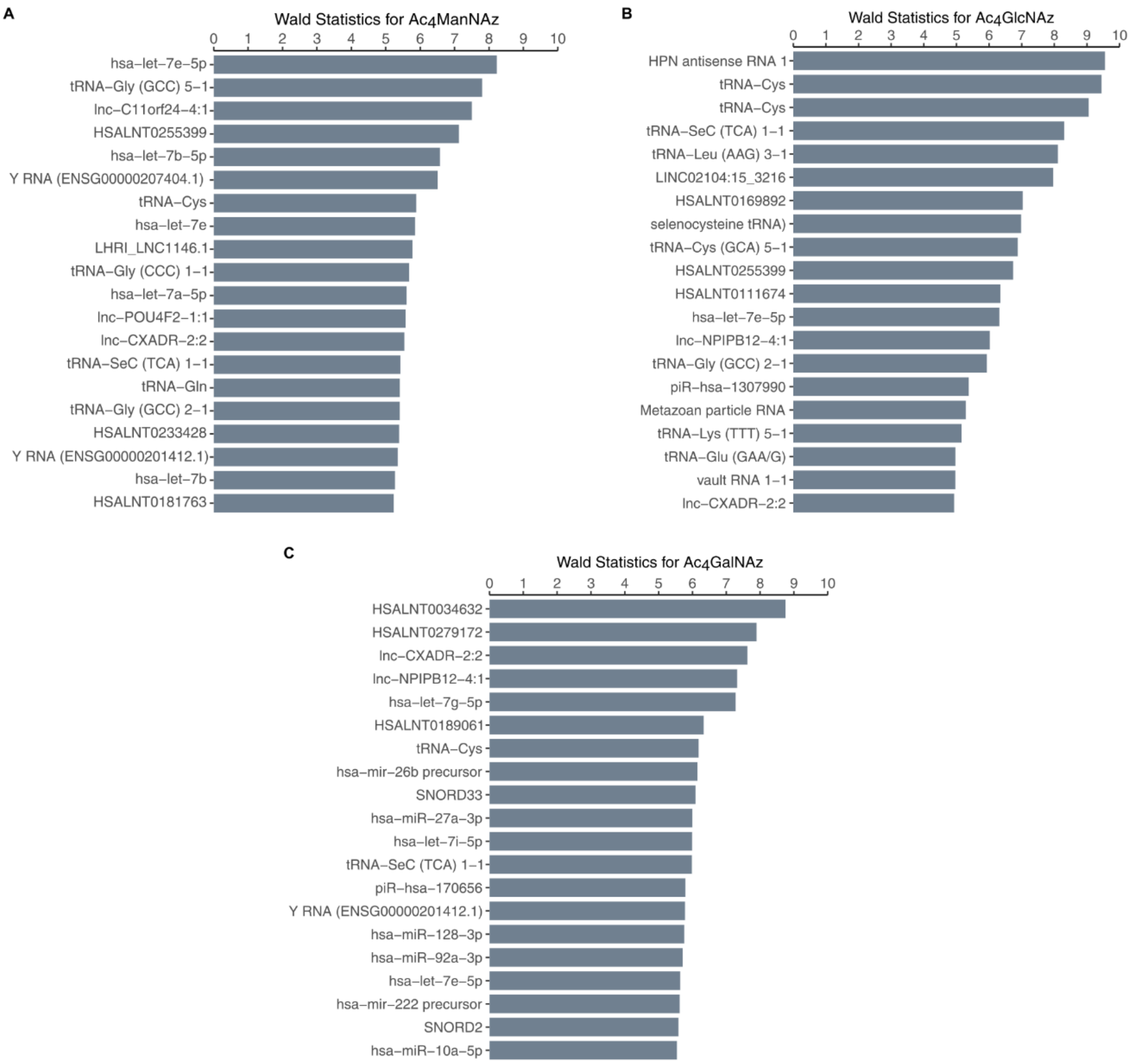

Fig. S7 Top ManNAz-, GlcNAz-, and GalNAz-RNA transcripts, their sequence motifs and GO terms.

(A) Top 20 of Ac_4ManNAz-enriched RNA with the highest Wald Statistics values listed in decreasing order. (B) Top 20 of Ac_4GlcNAz-enriched RNA with the highest Wald Statistics values listed in decreasing order. (C) Top 20 of Ac_4GalNAz-enriched RNA with the highest Wald Statistics values listed in decreasing order.

3. References

1. D. Fürniss, T. Mack, F. Hahn, S. D. L. Vollrath, K. Koroniak, U. Scheppers, S. Bräse, *Beilstein J. Org. Chem.* **2013**, *9*, 56–63.

2. D. Rabuka, S. C. Hubbard, S. T. Laughlin, S. P. Argade, C. R. Bertozzi, *J. Am. Chem. Soc.* **2006**, *128*, 12078–12079.
