# Supplementary material for: An Expanded View of RNA Modification with Carbohydrate-Based Metabolic Probes
Source: JACS Au. 2025 May 5;5(5):2309–20. doi: 10.1021/jacsau.5c00249 (PMC12117391; doi:10.1021/jacsau.5c00249)
Supplement: Supplementary file 3 [file au5c00249_si_003.pdf]

Table S2 Intersecting hits ManNAz-, GlcNAz-, and GalNAz-RNA

Name	Family	Ac4ManNAz			Ac4GlcNAz			Ac4GalNAz			Sum of stat	Mean of stat
		lf2c	padj	stat	lf2c	padj	stat	lf2c	padj	stat		
**hsa-let-7e-5p**	miRNA	4.294318087	1.92E-13	8.225666338	3.307983189	4.17E-08	6.317132485	2.935252882	2.61E-06	5.63863245	20.18143127	6.727143758
**tRNA-Gly (anticodon GCC) 5-1 (TRG-GCC5-1)**	tRNA	2.439954397	3.99E-12	7.802856537	1.047985699	0.014616909	3.344038747	0.960978666	0.036693829	3.075083582	14.22197887	4.740659622
**hsa-let-7b-5p**	miRNA	3.743655091	1.74E-08	6.57543182	1.675824658	0.038027555	2.944737129	2.678797744	0.000205879	4.692026283	14.21219523	4.737398411
**tRNA-Cys**	tRNA	3.337301614	8.76E-07	5.884761474	5.424012517	3.39E-18	9.443353018	3.493636686	1.81E-07	6.181074093	21.50918859	7.169729528
**tRNA-Gly (anticodon CCC) 1-1 (TRG-CCC1-1, TRG-CCC1-2)**	tRNA	3.972560771	2.57E-06	5.678019804	2.733728036	0.003122645	3.9033955	3.129108495	0.000428487	4.511590156	14.09300546	4.697668487
**non-protein coding lnc-CXADR-2:2**	lncRNA	6.376931266	4.80E-06	5.539484131	5.323131771	7.28E-05	4.931734351	9.067482584	1.47E-11	7.626526176	18.09774466	6.032581553
**tRNA-SeC (anticodon TCA) 1-1 (TRU-TCA1-1)**	tRNA	3.009853977	8.82E-06	5.424971936	5.472421972	4.95E-14	8.298334405	3.400531171	4.47E-07	5.982093004	19.70539935	6.568466448
**tRNA-Gly (anticodon GCC) 2-1 (TRG-GCC2 1 to 6)**	tRNA	4.185331441	9.00E-06	5.408123239	4.652575987	3.91E-07	5.930243608	3.427340799	0.000536612	4.439396355	15.7777632	5.259254401
**HSALNT0233428**	lncRNA	8.026667598	9.70E-06	5.388367199	5.749176274	0.005199541	3.741565422	7.011337058	7.32E-06	5.41362595	14.54355857	4.847852857
**Y RNA (ENSG00000201412.1)**	Y_RNA	3.319859764	1.14E-05	5.346401618	2.360354211	0.005414018	3.729495303	3.709051119	1.34E-06	5.777848105	14.85374503	4.951248342
**non-protein coding lnc-IL15RA-1:5**	lncRNA	21.32081461	3.92E-05	5.085661328	13.81967244	0.019107707	3.245850639	22.6837341	7.32E-06	5.413525756	13.74503772	4.581679241
**HSALNT0022915**	lncRNA	9.296224065	4.61E-05	5.044625697	5.708907456	0.026456101	3.094603462	6.483472306	0.01284345	3.509465401	11.64869456	3.882898187
**non-protein coding lnc-NPIPB12-4:1**	lncRNA	4.36572357	0.000335077	4.575834829	6.86564803	2.39E-07	6.022013751	8.900967267	1.31E-10	7.320820787	17.91866937	5.972889789
**hsa-miR-125a-5p**	miRNA	2.860542744	0.001297827	4.198226424	2.095914838	0.026456101	3.090941265	2.525265956	0.007632845	3.670821198	10.95998889	3.653329629
**small nucleolar RNA, C/D box 33 (ENSG00000199631.1)**	snoRNA	3.007875455	0.001534906	4.146527874	3.242589532	0.002851837	3.933875716	5.526390524	2.77E-07	6.093282136	14.17368573	4.724561909
**hsa-miR-98-5p**	miRNA	2.44085316	0.001950592	4.075803722	1.982607661	0.017888926	3.269534992	3.170547136	8.56E-06	5.380382519	12.72572123	4.241907078
**non-protein coding lnc-SPATA9-1:2**	lncRNA	7.622001637	0.002073553	4.057472073	6.561943427	0.011802558	3.429898106	8.483440338	0.000536612	4.438346712	11.92571689	3.975238964
**hsa-miR-320a-3p**	miRNA	2.962829053	0.00301288	3.930355135	2.693086393	0.010936776	3.460528427	2.6754506	0.021162336	3.323548949	10.71443251	3.571477504
**microRNA hsa-mir-423 precursor**	miRNA	3.650954894	0.004855622	3.782879812	2.767522386	0.048913731	2.827454749	3.396080284	0.033830197	3.12238769	9.732722251	3.24424075
**microRNA hsa-mir-30d precursor**	miRNA	2.122995441	0.009025661	3.575865342	1.717435846	0.042144278	2.898282182	2.289960881	0.005015425	3.794366789	10.26851431	3.422838104
**non-protein coding lnc-ABHD12-4:2**	lncRNA	4.958106781	0.015991831	3.375909469	6.358644548	0.003048436	3.914467899	7.556136501	0.000250514	4.640960241	11.93133761	3.977112536
**hsa-let-7g-5p**	miRNA	1.460971907	0.02482487	3.218251258	1.326409287	0.038662252	2.938641482	3.283696552	1.63E-10	7.27546921	13.43236195	4.477453983
**mir-320 microRNA precursor family**	pre_miRNA	6.073954578	0.024980101	3.214416708	5.592026512	0.042902524	2.889067892	7.173712022	0.006800809	3.705797555	9.809282155	3.269760718
**hsa-miR-382-5p**	miRNA	2.877623916	0.03359151	3.073684491	3.896822482	0.002135619	4.032455026	3.458808951	0.002736866	4.003966935	11.11010645	3.703368817
**mitochondrially encoded tRNA-Cys (UGU/C) (MT-TC)**	tRNA	5.635349197	0.0338735	3.068956151	6.67004193	0.012955286	3.388669484	6.626132057	0.019469925	3.352986686	9.810612321	3.270204107
**miR-HCC1**	miRNA	2.773293813	0.034006908	3.065252858	3.795189459	0.004236148	3.81225293	5.280167465	2.50E-05	5.155705591	12.03321138	4.01107046
